# Mechanoresponsive scatterers for high-contrast optical modulation

**DOI:** 10.1515/nanoph-2021-0642

**Published:** 2021-12-17

**Authors:** Donghwi Cho, Haomin Chen, Jonghwa Shin, Seokwoo Jeon

**Affiliations:** Department of Materials Science and Engineering, KAIST Institute for Nanocentury, Korea Advanced Institute of Science and Technology (KAIST), Daejeon 34141, Republic of Korea; Querrey Simpson Institute for Bioelectronics, Northwestern University, Evanston, IL 60208, USA; Department of Mechanical and Aerospace Engineering, The Hong Kong University of Science and Technology, Clear Water Bay, Kowloon, Hong Kong, P. R. China; Department of Materials Science and Engineering, Korea Advanced Institute of Science and Technology (KAIST), Daejeon 34141, Republic of Korea

**Keywords:** light scatterer, mechanochromic; nanocomposite, smart window, stretchable

## Abstract

Smart chromatic materials with optical transmittances that can be modified by light scattering upon external stimuli are attracting extensive interest because of their appealing applications in smart windows, privacy protection, electronic displays, etc. However, the development of these scatterers, which are mostly activated by electric fields, is hindered by their intrinsic energy consumption, slow responses, and poor stability. Recently, mechanoresponsive scatterers based on a strain-driven reconfiguration of the surface or internal structure have emerged, featuring fast responses and a simple composition/fabrication. Because there is no energy consumption to maintain the transparency/opacity, this novel scheme for scatterers holds great promise to break the existing bottleneck. This article presents recent advances in the development of mechanoresponsive scatterers and compares different structural design strategies. The scatterers are categorized into 2D, 3D, and other types according to the dimensions of their functioning structures. The fabrication methods, mechanisms, and relationships between the structural parameters and optical modulating performances are discussed for each category. Next, the potential applications of these scatterers are outlined. Finally, the advantages and disadvantages of the mainstream 2D and 3D categories are summarized, followed by a perspective on future research directions.

## 1 Introduction

**Figure 1:**
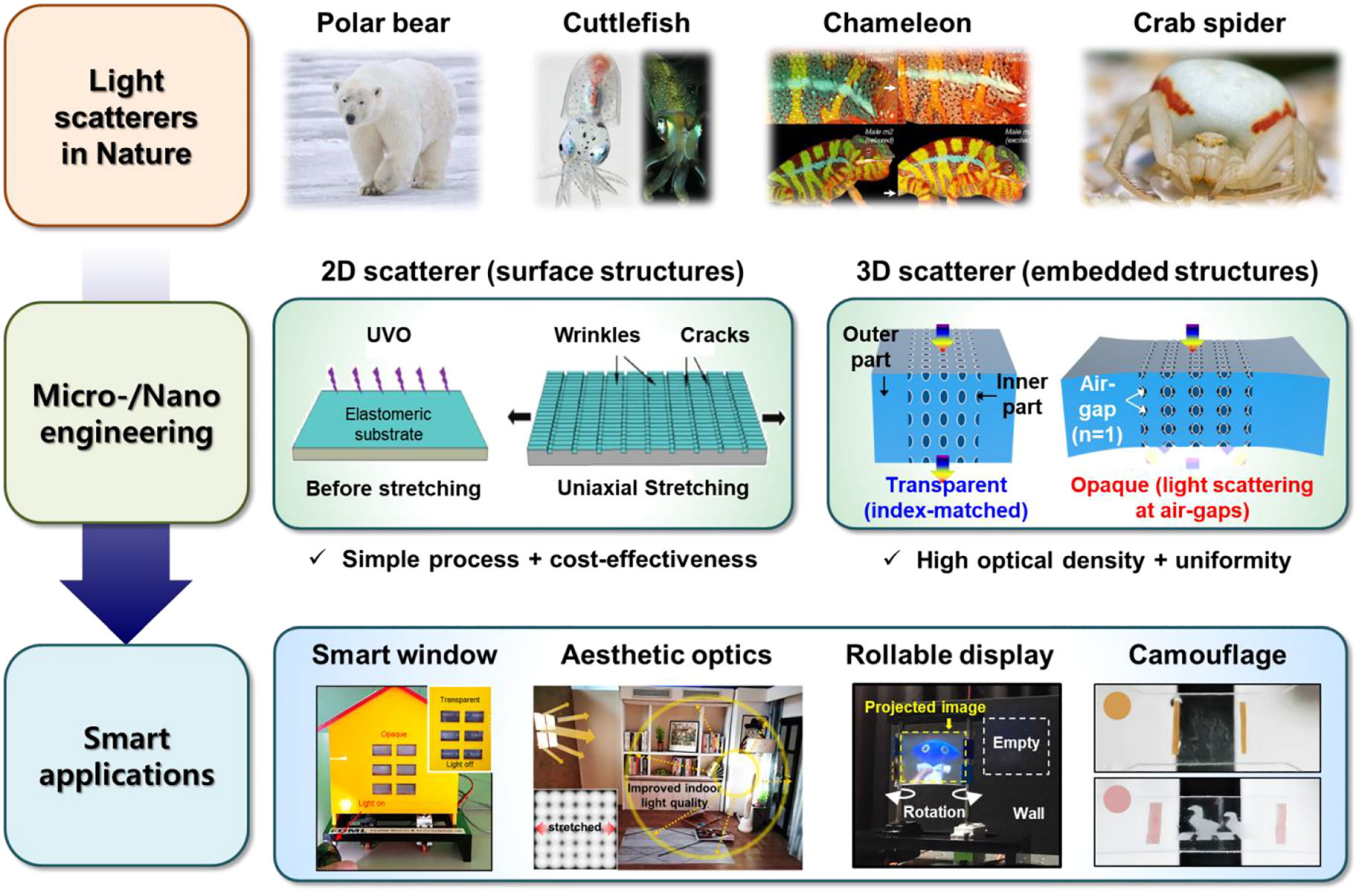
Schematic showing light scattering in bio-optics and optical engineering for smart applications. Reprinted with permission from Refs. [3, 17, 18, 89].

Light scattering is one of the physical processes associated with the visible appearance of most objects in the world, with the other being light absorption [1], [2], [3]. A pure white color and even remarkable color-changing features for the surface of objects owe their appearance to the dynamic control of visible light transmission by light scattering from internal and/or surface inhomogeneities such as texture, roughness, and refractive index mismatched components. Such compelling but natural characteristics have resulted in considerable recognition of the value of the optical scattering phenomenon. Specifically, as shown in Figure 1, scattering plays a versatile role in optical properties, as seen in many examples of natural scatterers, including i) the fur of polar bears, which keeps the animal warm and serves as camouflage by appearing to have a white color as a result of the scattering of infrared light and visible light, respectively [4]; ii) the transparency-changing/color-changing ability of cuttlefish in the sea [5]; iii) the tunable color and dynamic environmental adaption skills of a chameleon [6]; and iv) the highly pure white color produced by the disordered cube-like guanine crystals in gonocytes [7].

Inspired by such bio-optics, extensive technical developments have established a basis for controlling light scattering, which results in changes in the specular transmittance through a scatterer in response to external stimuli, including an electrical field [8], [9], [10], the temperature [11], [12], [13], and light [14], [15], [16]. Specular transmittance or normal transmittance refers to the transmitted portion of the incident light that is not scattered and is important because it determines how well the spectral information of the light is maintained. Although their high-contrast optical switching performances have already been commercialized, the chemical/mechanical instability, long response time, and fabrication complexity have been considered to be bottlenecks for their practical usage [8]. To overcome these technical hurdles, a new optical modulation scheme based on structural modifications of the scatterer in response to simple mechanical deformations has emerged in the form of “mechanoresponsive scatterers.” The well-known principle of strain-induced transmittance modulation is used to control the shape and size of the scattering sites on the surface or embedded in the material to be formed or eliminated [17]. With the reversible, strain-induced deformation and recovery of the scattering sites, the hybrid composite allows effective control of the scattering degree, leading to a change in color or optical transparency. Figure 1 summarizes two representative examples of scatterers that depend on different structural dimensions [17, 18], along with their advantages and potential applications. Because of its ability to reversibly control optical features, strain-induced light modulation is of great interest for various high-value-added applications, including smart windows, privacy protection, energy savings, electronic displays, and camouflage techniques [8].

**Figure 2:**
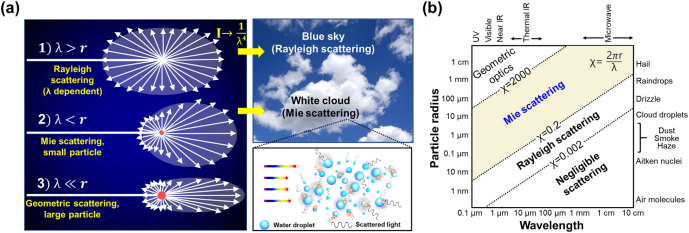
Dynamically tunable light scattering. (a) Light scattering dependence on the size of the scattering particle and associated examples. (b) Scattering regime dependence on the wavelength of the incident radiation and the particle radius of the scatterer.

Light scattering regimes can be theoretically subdivided into three domains [19, 20] on the basis of a dimensionless size parameter, *χ*, which is defined as follows:

(1) $$\chi =\frac{2\pi r}{\lambda }\text{,}$$

where *r* is the radius of a spherical scatterer and *λ* is the wavelength of the incident radiation through the medium (Figure 2a). Based on the value of *χ*, each domain is expressed as follows:

1. *χ* ≪ 1: Rayleigh scattering (small particle compared to the incident wavelength);
2. *χ* ≈ 1: Mie scattering (particle of approximately the same size as the incident wavelength, valid for spherical scatterer);
3. *χ* ≫ 1: geometric scattering (particle much larger than the incident wavelength).

Rayleigh scattering is a process involving the scattering of electromagnetic radiation (including light) by a small scatterer with a refractive index different from the environment (*i.e.*, a particle, bubble, droplet, and/or a density fluctuation in the gas phase) [21]. In other words, the radius of the scattering site is typically smaller than 1/10 of the wavelength of the incident light (approximately <50 nm in the case of visible light). The most well-known example of Rayleigh scattering is the blue color of the sky, which clearly demonstrates the strong wavelength dependence of the scattering cross section in the Rayleigh scattering regime. To control light scattering more uniformly over the entire visible light spectrum and achieve tunable optical transparency, larger scatterers are desirable.

The electromagnetic scattering from larger particles (with diameters of approximately 50 nm to several micrometers) is commonly known as Mie scattering, where the particle size is typically between 1/10 *λ* and *λ* [22], [23], [24]. While deep-subwavelength particles in the Rayleigh regime possess scattering cross sections that are orders of magnitude smaller than their physical cross sections, the two cross sections for wavelength-scale particles are comparable to each other in the Mie regime. Hence, for the same cumulative volume of scatterers, light is much more strongly scattered in the Mie regime. Moreover, in the presence of particle size inhomogeneity, the cross section is not strongly affected by the wavelength. This is why many clouds appear white: all the visible wavelengths experience multiple Mie scattering from water droplets in the cloud. Geometric scattering occurs when the ratio of the particle diameter to the incident wavelength is approximately 10 (approximately >1 µm), which is the general case of optical scattering where the light and particle interact [25, 26]. According to the classifications, the scattering regimes based on the ratio between the incident wavelength and the scattering site radius are summarized in Figure 2b. Considering the size-dependent scattering efficiency, the capability of controlling the scattering site size has been proven to provide many design opportunities for eye-detectable optical modulation, from transparency to opaqueness. Such intriguing features, in particular, can be transferred to practical technologies by means of scattering sites with sizes tunable by mechanical deformation.

To date, many review papers have summarized the rapid development of stimulus-driven scattering devices with deformable scattering sites for transmittance modulation. Nevertheless, the majority of these reviews focused on the well-established electro- [27], [28], [29], thermo- [11, 30, 31], and photoresponsive devices [32], while omitting the more recently developed topic of mechanoresponsive schemes. In the last decade, there has been intensive growth of interest in research on mechanoresponsive scatterers, ranging from the relatively more developed ones utilizing dynamic 2D surface features to the newly evolving ones based on the control of 3D heterogeneous interfaces [17, 33, 34]. It is imperative to summarize the fabrication methods, working mechanisms, structural effects on the mechano-optical behaviors, and emerging applications of mechanoresponsive scatterers to provide guidelines for developing next-generation mechanochromic devices and flexible displays. To address this gap, this review considers the recent progress in mechanoresponsive scatterers. The first two sections summarize the mainstream strategies for designing scatterers based on tunable 2D and 3D structures. Their respective fabrication methods and physical mechanisms are described in detail, and the structural factors that are essential to their final optical modulating performances are emphasized. In addition, the developed methodologies and functionalities involved in 3D scatterers are highlighted. The third section introduces other design strategies that provide more possibilities for the field. Fourth, the promising applications of mechanoresponsive scatterers are discussed. Finally, the advantages and disadvantages of 2D and 3D scatterers are comprehensively compared, and the outlook for future research to develop high-performance scattering materials is provided. The term “transmittance” refers to specular transmittance unless otherwise stated.

## 2 Optical modulation based on two-dimensional (2D) topographies

2D scatterers refer to optical films that dynamically modulate their transparency through tunable light scattering behaviors as a result of a change in the 2D surface topography. Since Stafford et al. reported light scattering by periodic wrinkles [35], extensive studies have been carried out to explore a variety of 2D surface features that are tunable for dynamic optical modulation [27, 30, 32, 36]. This section reviews the recent progress in developing 2D scatterers from the standpoints of the fabrication, working mechanism, and structural factors related to the optical modulation performance.

### 2.1 Fabrication of 2D scatterers

#### 2.1.1 Wrinkles induced by compression and modulus mismatch

**Figure 3:**
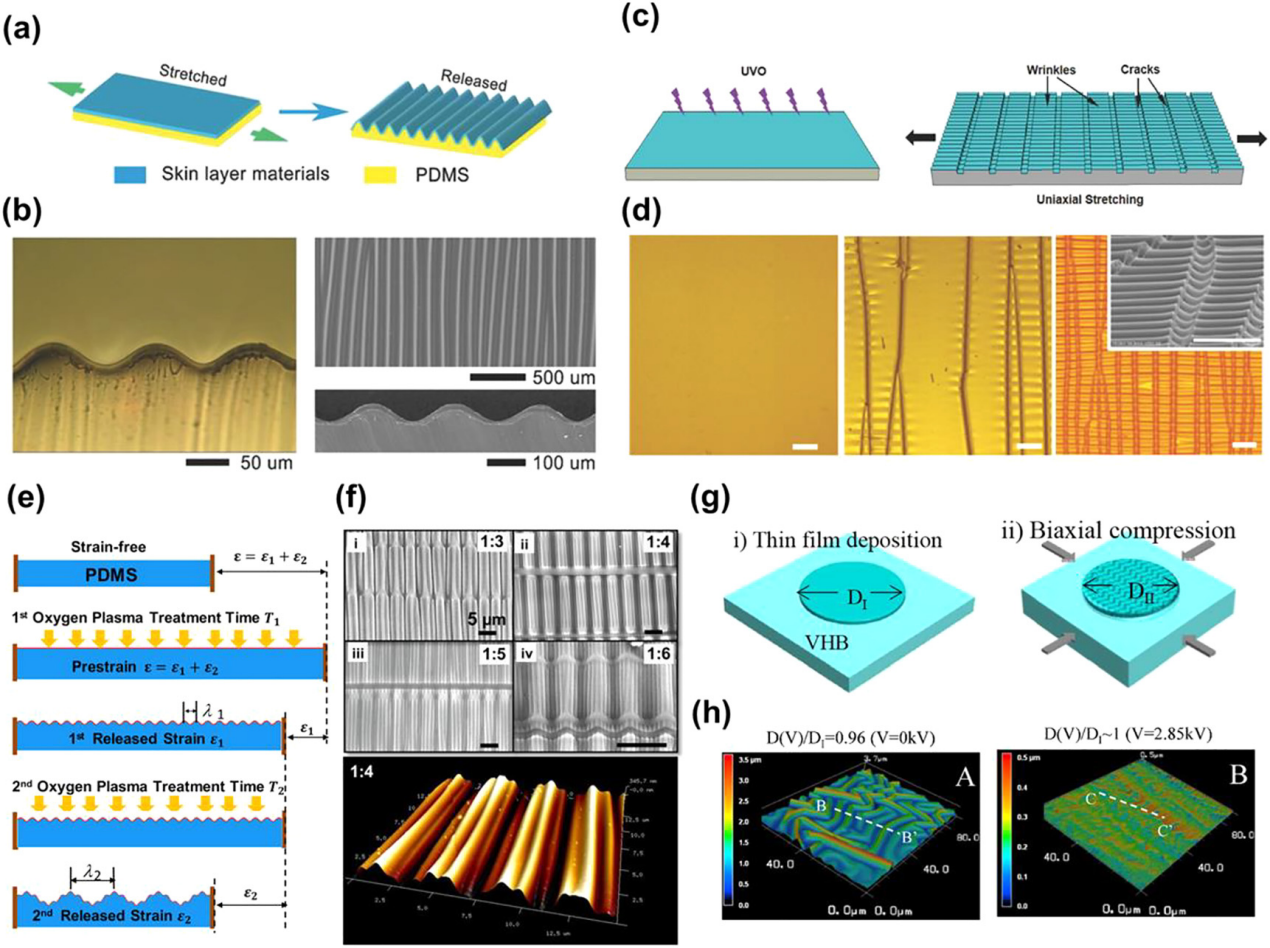
Fabrication of 2D scatterer based on surface wrinkles. (a) Schematics of 1D wrinkled film fabricated by pre-stretch/release process. (b) Optical microscope and SEM images of the as-prepared wrinkled PVA/PDMS film. (c) Fabrication processes and reversible tuning of optical transmittance of a PDMS film with surface wrinkling–cracking patterns. (d) Optical microscopy images of the PDMS film surface under various applied strains (scale bar: 200 µm). (e) Schematics of the fabrication process to generate hierarchical 1D wrinkles on PDMS through two-step strain release and two-step oxygen plasma treatment. (f) SEM images of hierarchical wrinkles with different wavelength ratios and AFM images of 1:4 hierarchical wrinkles. (g) Fabrication procedures for 2D wrinkles from thin-film deposition to radial compression-induced surface wrinkling. (h) Confocal micrographs showing the morphology of the as-prepared thin film in 2D wrinkled and unfolded states. Reprinted with permission from Refs. [18, 57, 60, 61].

Wrinkles are a typical type of surface texture that can be dynamically tailored by mechanical strains and have been extensively explored for use in stretchable electronics [37], [38], [39], optical devices [32, 40], [41], [42], [43], [44], [45], energy storage devices [46], and mechanical property characterizations [47], [48], [49]. The curved surface of a wrinkle refracts the incident light to different directions depending on the incident angles, blurring the objects behind the wrinkled thin layer/substrate bilayer film. However, when the wrinkles are flattened by lateral tensile strains, light beams pass through the film with minimal deflection, and the film turns from opaque to translucent to highly transparent. Wrinkles are mostly formed by the prestrain-release process (Figure 3a and b). After depositing a relatively stiff material onto a uniaxially prestretched elastomer or viscoelastic substrate, 1D wavy wrinkles are formed when the prestrain is released because of the induced compression and modulus mismatch between the thin layer and substrate [18]. The methodologies for preparing the top thin layer include transferring [50, 51], deposition [52, 53], coating [18, 54], and surface reactions [33, 55], [56], [57]. In addition to mechanical strain release, compressive strain can also be generated by increasing the temperature over the glass transition temperature of a prestrained shape memory polymer substrate or cooling a preheated substrate that has a higher thermal expansion coefficient than the thin layer on top [58, 59].

The majority of wrinkle-based scatterers change from opaque to transparent when lateral strain is applied; however, this is not the only possible method. By simply applying ultraviolet ozone (UVO) plasma to a polydimethylsiloxane (PDMS) substrate, Li et al. fabricated a silica/PDMS bilayer film with high transparency in the released state but high opacity in the stretched state (Figure 3c) [60]. This was because of the featureless surface of the as-prepared film, which allowed a high transparency of 92%, similar to that of the intrinsic PDMS film. When the bilayer film was stretched to strains beyond 5%, cracks formed along the direction transverse to the stretching direction owing to the brittleness of the top silica layer (Figure 3d). Meanwhile, compression was generated due to the high Poisson’s ratio of PDMS (∼0.5), which induced the wrinkling of the silica/PDMS film when it exceeded the critical buckling strain. Both cracks and wrinkles contributed to light scattering, endowing the scatterer with a low transmittance of 9.2% at 50% strain. Using a similar approach, Zeng et al. designed a bilayer film with a polyvinyl alcohol (PVA)/laponite rigid film strongly bonded to a soft PDMS substrate [32]. Based on reversible cracking and wrinkling, the bilayer film exhibited a transmittance contrast of ∼55% at 50% strain.

In addition to the conventional 1D wrinkled structure, scatterers with multilevel 1D wrinkled structures have been proposed to simultaneously achieve structural color and transmittance modulation for reflection enhancement [52, 57]. Lin et al. reported a two-step oxygen plasma treatment–strain release process, which generated multilevel 1D wrinkles with different wavelengths and periods (Figure 3e). The wavelength ratio between the larger and smaller wrinkles was tailored from 1:6 to 1:3 by controlling the combination of the sequential release of prestrain and the plasma treatment time (Figure 3f). When the strain was applied, it was found that the large wrinkles on the microscale were flattened, causing the opaque film to become transparent, while the small wrinkles on the nanoscale were maintained, resulting in a nonvanishing structural color throughout the deformations. The angle-dependent structural color originated from the Bragg diffraction by the periodic wavy nano-wrinkles, which, on the other hand, limited the maximum transmittance to ∼75% at a light wavelength of 550 nm. Such limitations of multilevel 1D wrinkles can be overcome by increasing the wrinkle dimensionality to 2D. To fabricate 2D wrinkles with a more complicated texture, TiO_2_ was deposited onto a radially prestrained VHB tape [61]. Utilizing the mechanical instability of the surface, 2D wrinkles were formed when the prestrain was released (Figure 3g). The light was effectively scattered by the wrinkles, giving a low transmittance of ∼2% under a low precompression of 4% strain. The transmittance increased significantly to ∼80% once the wrinkles were unfolded (Figure 3h). A 2D wrinkled ZnO/VHB film prepared by a similar methodology exhibited an exceptional 90% transmittance contrast when the 14% radial compression was released. Interestingly, such radial deformation characteristics cause a scatterer based on 2D wrinkles to be well matched with the technology for dielectric elastomer actuators [61, 62]. By fabricating a conductive coating as stretchable electrodes on the top and bottom of the elastomer substrate, optical modulation of the scatterers can be activated within seconds by an applied voltage that vertically compresses and, in turn, laterally elongates the substrate by electrostatic pressure, which flattens the wrinkles.

#### 2.1.2 Fabrication of hierarchical and random surface features

**Figure 4:**
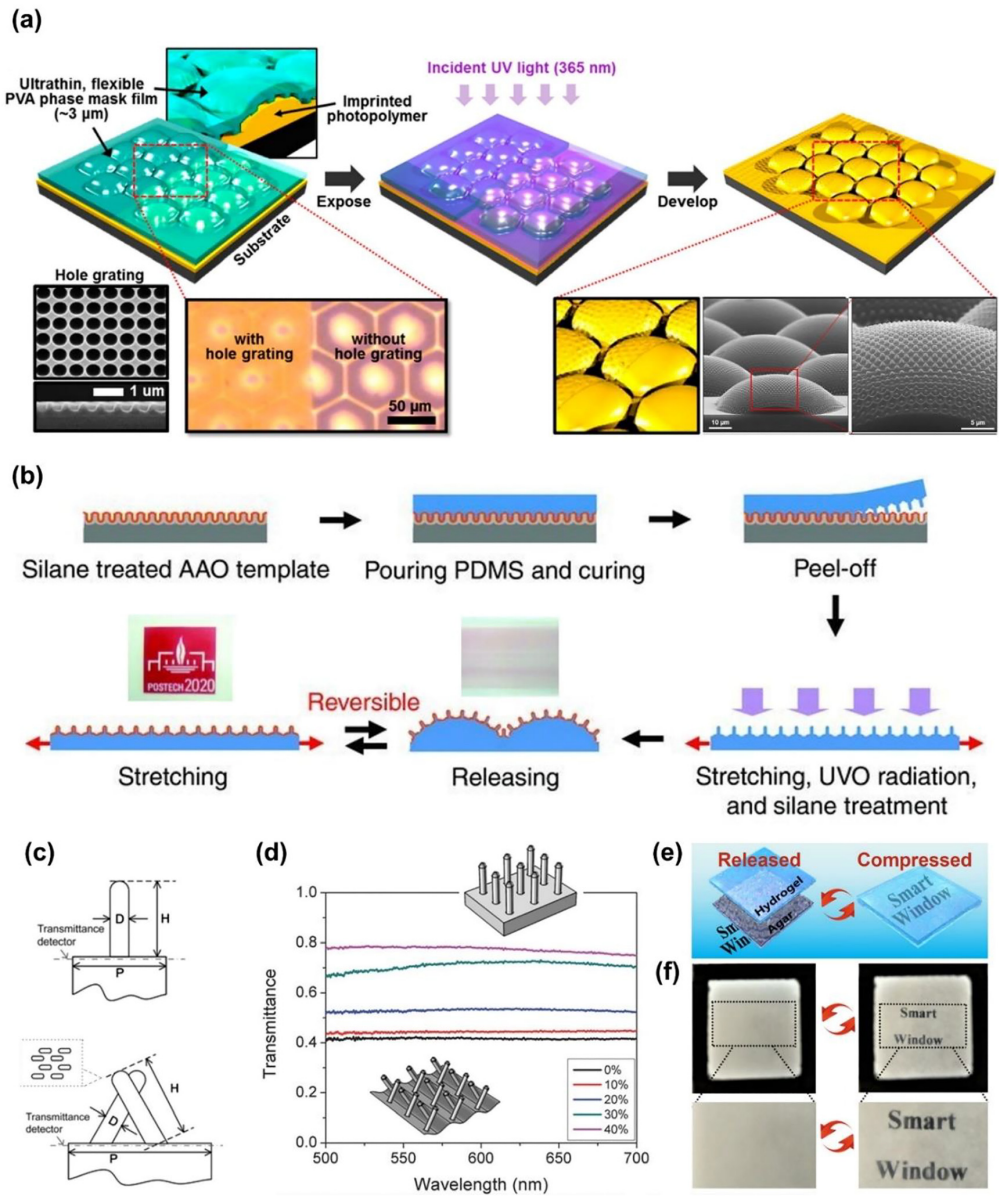
Fabrication of 2D scatterers based on random and hierarchical surface features. (a) Schematics describing flexible near-field phase-shift lithography (NFPSL) to fabricate nanostructure on a wavy substrate. (b) Fabrication processes for an optical thin film with hierarchical surface features, including replica molding, surface wrinkling, and treatment of the PDMS film. (c) Schematic illustration of pillar features in straight and tilted states. (d) Transmission spectrum of the hierarchical PDMS film with a wrinkled pillar array at different strains. (e) An agar/hydrogel pressure-responsive smart window showing opaqueness under release and transparency under compression. (f) The corresponding digital photos showing the optical modulation of the smart window. Reprinted with permission from Refs. [33, 63, 67, 68].

Nanopatterning, including photolithography, nanoimprinting, and soft lithography, has been widely adopted to fabricate delicate hierarchical surface patterns for various optical properties. As shown in Figure 4a, a “nano-on-micro” hierarchical photonic structure mimicking a moth’s eye was prepared by combining different photolithography technologies [63]. A hexagonal array of photoresist microdomes was fabricated using conventional photolithography and imprinting. Flexible near-field phase-shift lithography (NFPSL) was used to generate nanopores on top of these microdomes. An ultrathin PVA phase mask consisting of hole gratings was used to generate a periodic Talbot image inside the microdome-shaped photoresist upon exposure. The low thickness allowed conformal contact between the phase mask and the curved surface, which guaranteed the regularity of the exposure light distribution and thus the nanoporous structure [64, 65].

Compared to microscale wrinkle engineering, nanopatterning has the advantage of high accuracy at the nanoscale and a large variety of feature geometries. Therefore, many efforts have been made to utilize this accurate but costly technique as a supplement for wrinkle engineering to fabricate different hierarchical surface structures for multifunctional applications. Lee et al. first prepared PDMS with nanopillar arrays replicated using a nanoporous anodic aluminum oxide template, and then generated 1D wrinkles through the prestrain-release process (Figure 4b). The resultant structured PDMS film exhibited the reversible modulation of its transparency and wetting properties and could be used as a smart window capable of self-cleaning and antireflection. However, it is more complicated to combine microscale pillars with wrinkles for enhanced transmittance tunability because the pillars greatly alter the stress relief upon releasing the prestrain [66, 67]. PDMS with its surface patterned with a pillar array was replica molded from a master. Micropillars tilted by the underlying wrinkles were realized by carefully designing the thickness of the silica top layer and the prestrain magnitude/direction [67]. The hierarchical structure of these tilted pillars on the wrinkles increased the film opacity (Figure 4c), but unfortunately, it also decreased the transmittance when the wrinkles were flattened and the pillars were straightened. Therefore, a moderate transmittance contrast of ∼40% was achieved for the hierarchically structured film, which was only 10% higher than that of the film with bare wrinkles (Figure 4d). Yet the straightened pillars on wrinkles offered more effective color gratings than bare wrinkles, and the resultant smart window exhibited three optical states: opaque, colored, and transparent.

In contrast to hierarchical structures, random surface features can easily be fabricated using simple and cost-effective processes. On the other hand, random features are also effective in light refraction. An agar/hydrogel smart window was reported by Wang et al., which showed a transmittance of 30% in the opaque state due to the roughness of the agar surface (with a root mean square roughness, *R*_*a*_, of 315 nm) that spontaneously formed during water evaporation (Figure 4e) [68]. The light scattering at the agar/air interface was significantly reduced when the surface features conformally contacted to the hydrogel by compression, revealing the originally “hidden” words behind the smart window (Figure 4f).

### 2.2 Optical modulation mechanism of 2D scatterers

**Figure 5:**
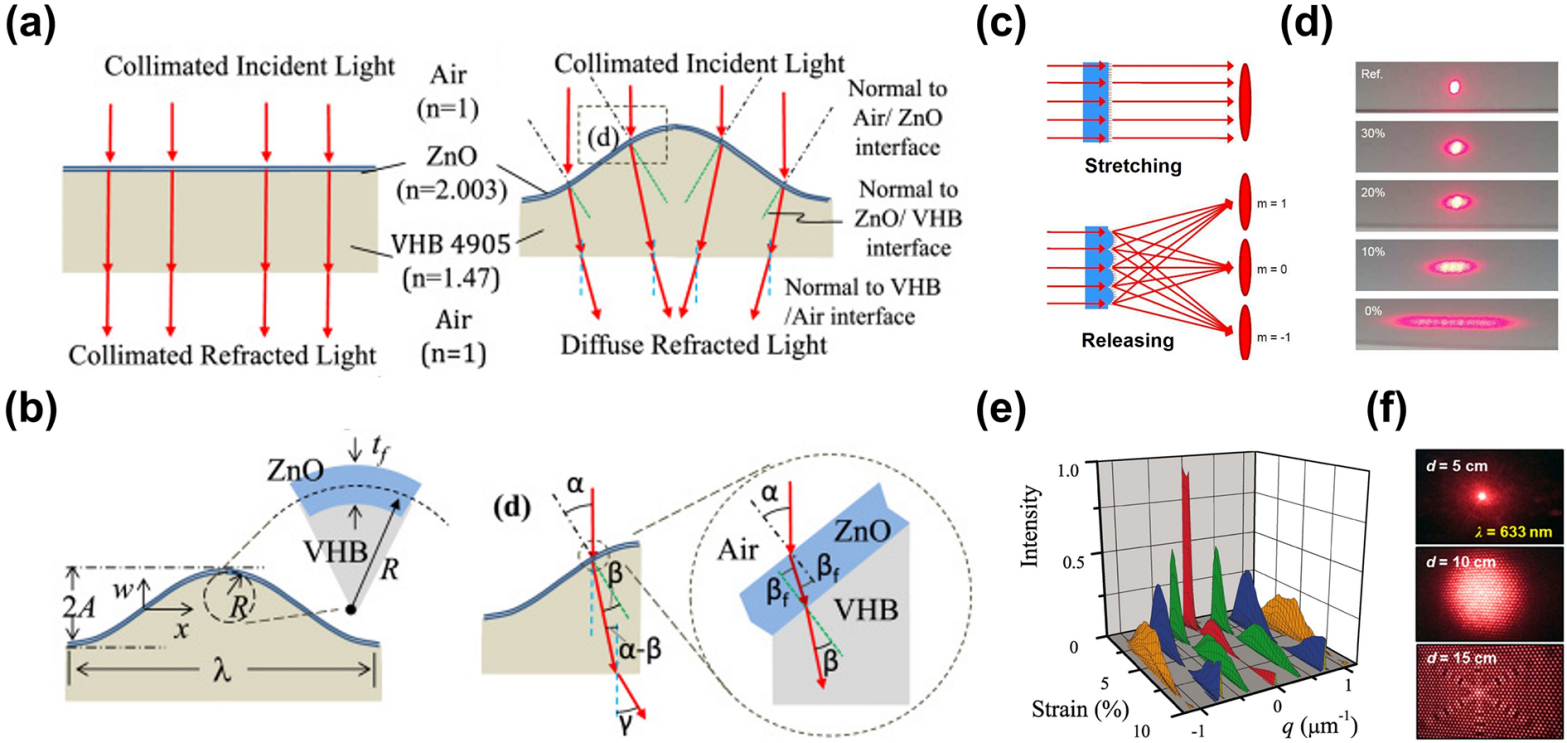
Mechanisms of dynamic optical modulation by 2D scatterers. (a) Schematics of light refraction by a flat and wrinkled bilayer composite. (b) A model of unidirectional wrinkles with a sinusoidal profile and the multiple refractions of a ray of light through the wrinkled surface. (c) Schematic illustration of light transmission through a periodic wrinkled film under stretching and release. (d) Diffracted beams produced when laser light passes through a microwrinkled elastomeric film stretched to different strains. (e) Normalized *I*/*I*_0_ data as a function of compressive strain where the zeroth, first, second, and third orders are indicated in red, green, blue, and orange, respectively. (f) Diffraction and diffusion of incident light (*λ* ∼ 633 nm) passing through an optical film with hierarchical 2D surface patterns as a function of observation distance. Reprinted with permission from Refs. [33, 63, 69, 70].

Consider the refraction of visible light through a surface with a single micro-wrinkle in the field of geometrical optics. In the flattened state (Figure 5a), light rays are normally transmitted through a horizontal optical film consisting of a thin top layer on an elastomer substrate and continue in the same direction. However, when incident onto a wrinkled surface, the light is refracted to different directions depending on the local incident angle and the refractive index mismatch between the air and the film compositions [69]. For a light ray moving in the vertical direction incident to a sinusoidal wrinkle with incident angle *α* (Figure 5b), there are three light refractions at the air/thin film, thin film/substrate, and substrate/air interfaces. Because the top layer usually has a low thickness of up to tens of nanometers [51, 69], [70], [71], its curvature is negligible. The refracted angle at the first interface is equal to that at the second interface. The angle of the third refraction, *γ*, is related to *α* according to Snell’s law:

(2) $$\gamma =\sin^{-1}\left(\sin\;\alpha \cdot \sqrt{{n_{substrate}}^{2}-{\sin\;}^{2}\;\alpha }-\frac{1}{2}\sin\;2\;\alpha \right)\text{,}$$

where *n*_*substrate*_ is the refractive index of the substrate. Given that *α* varies from 2*πA*∕*D* to −2*πA*∕*D* over a half wavelength of the wrinkle, where *A* and *D* are the amplitude and wavelength, respectively, a higher aspect ratio, *A*∕*D*, leads to a larger refracted angle deviation from the normal direction and thus a better light scattering performance for a single sinusoidal wrinkle.

A group of multiple periodically positioned wrinkles can be considered as an optical grating, especially when the wrinkle periodicity is comparable to or less than the light wavelength [36, 72]. As light passes through a 1D wrinkled surface, its phase is shifted by a magnitude proportional to the wrinkling amplitude. Diffraction patterns can be observed when the incident light is coherent (Figure 5c and d) [33], and its intensity can be calculated, in the Fraunhofer limit, as [70]:

(3) $$I\left(q\right)\approx \sum_{p=-\infty }^{\infty }J_{p}^{2}\left(\frac{m}{2}\right){\text{sinc}}^{2}\left[\frac{W}{\pi }\left(q-\frac{2p\pi }{D}\right)\right]\text{,}$$

where *J*_*p*_ is the Bessel function of the first kind, *m*/2 is the maximum phase change of the light after passing through the wrinkle, *W* is the half-width of the aperture for the light source, *p* is an index indicating the diffraction order, and *D* is the wavelength of the wrinkle. Because *m* is a monotonic function of the compressive strain, the diffraction orders in Figure 5e follow the Bessel function with only a slight distortion. The change in intensity is not monotonic, but cyclic through its maxima and minima as the wrinkle amplitude increases with increasing compressive strain. When the wrinkle dimensionality increases to 2D, the diffraction patterns also extend to become complex throughout the 2D plane with high diffraction orders (Figure 5f) [63, 73], causing the light to be highly diffused after passing through the optical film [71].

### 2.3 Structural factors in scattering performance

Despite similar fabrication approaches, the existing mechanoresponsive scatterers in the literature present optical modulations that differ greatly from one another. The scattering performance of a scatterer is determined by multiple structural factors for the surface features. This section discusses the influence of four structural factors, including the bonding of thin film/substrate interface, amplitude and wavelength of the wrinkles, cracks, and wrinkle orientation. Their relationships with the fabricated surface morphology and transmittance modulating capability are systematically analyzed to provide insights into the origins of different scattering behaviors and guidelines for designing scatterers based on 2D surface features.

#### 2.3.1 Interfacial bonding

**Figure 6:**
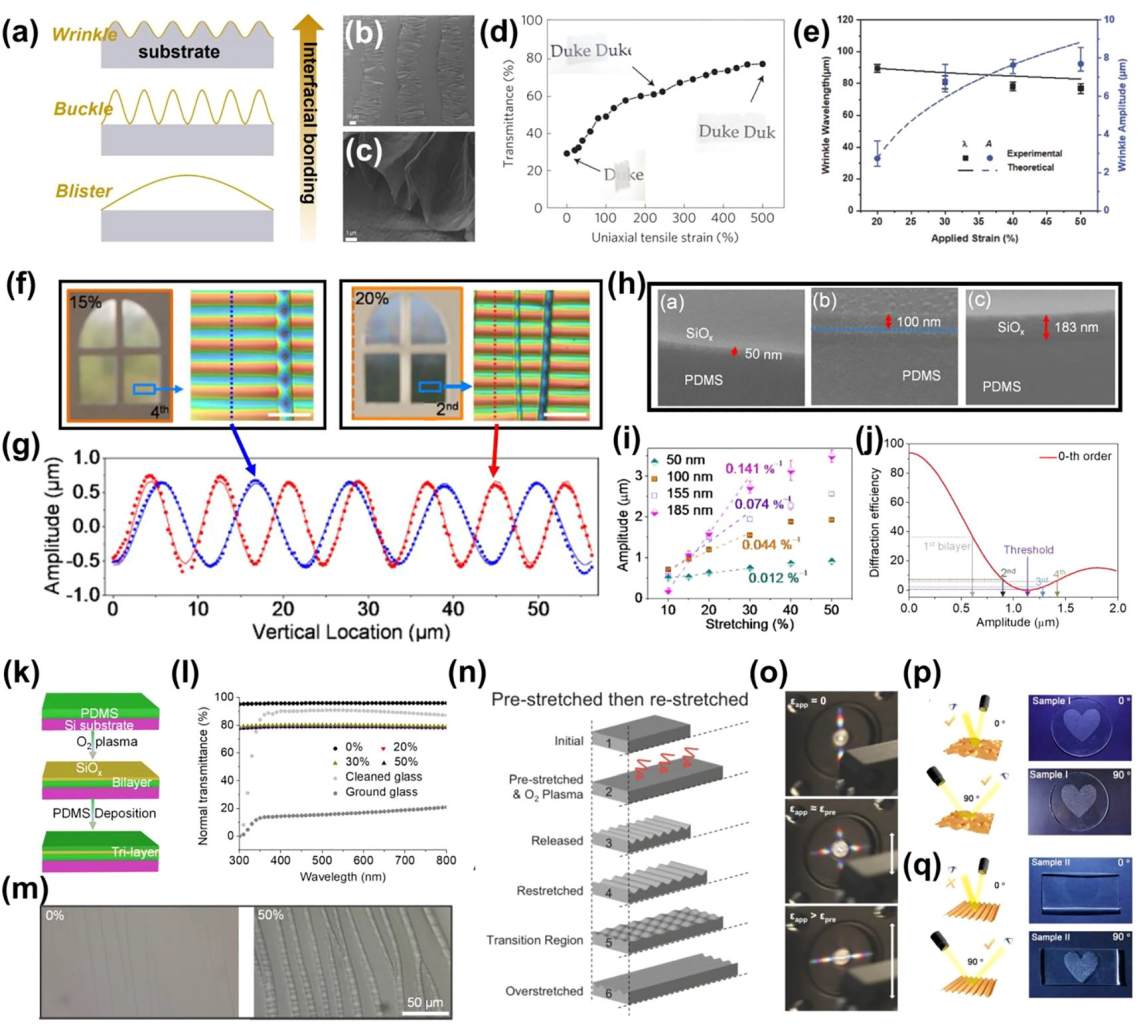
Structural factors for 2D scatterers. (a) Schematics showing the formation of wrinkles, buckles, and blisters according to the interface interaction between the stiff top layer and substrate. (b) SEM images of a typical delamination buckling pattern prepared by releasing a uniaxial strain of ≈400%. (c) High-resolution SEM image of an individual blister showing sharp folds. (d) Average normal transmittance as a function of the tensile strain applied to the buckled bilayer. (e) Wrinkle wavelength and amplitude as a function of different applied compressive strains. (f) Transparency and microstructures of wrinkled bilayer films at tensile strains of 15 and 20%, respectively, showing the same wrinkle amplitude but different wavelengths. (g) Corresponding vertical line cut profiles of the microstructures. (h) Cross-sectional SEM images of the SiO_*x*_ layers generated by 10, 30, and 90 min of UVO treatment. (i) Wrinkle amplitude as a function of the applied tensile strains for bilayer films with different stiff top layer thicknesses. (j) The dependency of the 0th order diffraction efficiency on the wrinkle amplitude. (k) Schematics showing the fabrication of a PDMS/SiO_*x*_/PDMS tri-layer film without wrinkles. (l) The transmittances of the tri-layer film under various tensile strains. (m) Confocal laser microscopy images of crack evolution in tri-layer film. (n) Schematics illustrating the fabrication of a 1D wrinkle and its evolution during re-stretching. (o) Wrinkled PDMS acting as gratings to diffract white light along different axes according to the wrinkle orientation. (p) Digital images of a heart-shaped pattern on a sample with 2D random wrinkles, showing the isotropic light scattering behavior. The light is incident from two directions perpendicular to each other. (q) Anisotropic scattering behavior of sample with 1D wrinkle. Reprinted with permission from Refs. [51, 56, 60, 71, 76, 82].

Depending on the interfacial bonding between the top thin film and the substrate, the same prestraining-releasing process results in wrinkles or delamination buckles in the bilayer structure (Figure 6a). Wrinkling occurs under compression when the substrate deforms with the top thin film without debonding. On the other hand, the film buckles up when its interfacial interaction with the substrate is relatively weak; thus, the two layers are partially and locally detached [71], resulting in periodic delamination buckling with a high aspect ratio (Figure 6b and c). It should be noted that if the interaction is too weak, one large blister instead of periodic buckles will be generated under compression (Figure 6a) [71]. When the interface bonding is relatively strong, delamination buckling does not occur instantly once the film is compressed, but gradually occurs in the form of fatigue failure. By controlling the UVO treatment time and thus the adhesion energy between the PDMS substrate and the semiconductor polymer thin layer, Sun et al. compared the buckling behaviors of samples undergoing cyclic strain [74]. It was found that the top layer did not buckle immediately after the first strain cycle in a sample with a medium interface. However, as the cyclic strain gradually weakened the bonding, delamination buckling was observed. On the contrary, samples with a high interfacial adhesion energy exhibited a negligible change in surface roughness even after 50 strain cycles.

Similar to the wrinkles, the buckles can be flattened and reappear reversibly as lateral tensile strains are applied or removed [51], endowing the bilayer structure with tunable optical properties. Zang et al. applied a graphene/acrylic bilayer film for dynamic optical modulation [51]. Because of the weak interaction between the graphene and acrylic substrate, the few-layer graphene buckled when the biaxial prestrain was released uniaxially and crumpled when the substrate was biaxially relaxed. The bilayer film exhibited transmittances of 30 and 80% in the opaque and transparent states, respectively, but such a 50% contrast required an impractically large uniaxial strain of 500% (Figure 6d). Similarly, Thomas et al. reported a high transmittance contrast of 80% in a graphene oxide/silicone scatterer with 2D buckles, which was activated by a 400% biaxial tensile strain. Compared to wrinkle-based scatterers, scatterers based on delamination buckles generally exhibit low opacity in the opaque state, leading to limited achievable optical contrast. From Eq. (2), it can be qualitatively shown that a high refractive index for the material below the thin film plays an important role in the scattering performance. The empty space under the buckles constrains the maximum opacity and overall transmittance contrast. Therefore, the high-contrast optical modulation of buckle-based scatterers, if possible, requires ultra-large activation strains, making delamination buckling a detrimental factor to the scattering performance.

#### 2.3.2 Wrinkle amplitude and wavelength

The wrinkle morphology is dependent on the compressive strain applied to the bilayer structure, according to the well-established finite-deformation buckling theory. When the compliant substrate thickness is more than 10 times larger than the stiffer thin film on top, the wavelength and amplitude of the wrinkle can be expressed as follows [50, 75]:

(4) $$D=\frac{2\pi h_{f}}{\left(1+\epsilon_{comp}\right){\left(1+\xi \right)}^{\frac{1}{3}}}{\left(\frac{\bar{E_{f}}}{3{\bar{E}}_{s}}\right)}^{1/3}\text{,}$$

(5) $$A=\frac{h_{f}}{\sqrt{1+\epsilon_{comp}}{\left(1+\xi \right)}^{1/3}}\sqrt{\frac{\epsilon_{comp}}{\epsilon_{c}}-1}\text{,}$$

where *h*_*f*_ is the thickness of the top thin film; *ε*_*comp*_ is the compressive strain applied to the bilayer structure; ${\bar{E}}_{f}$ and ${\bar{E}}_{s}$ are the plane strain moduli of the thin film and the substrate, respectively, which are determined by the Young’s modulus and Poisson’s ratio in each case; *ε*_*c*_ is the critical buckling strain determined by the ratio between the two plane strain moduli; $\xi =\left(5/32\right)\epsilon_{comp}\left(1+\epsilon_{comp}\right)$ demonstrates the effect of a large deformation and the geometrical nonlinearity of the substrate. The morphological changes in the wrinkles are reflected in the changes in the amplitude and wavelength. Generally, as the compressive strain increases, the amplitude increases, and the wavelength decreases, resulting in strong light scattering and opacity for the bilayer film (Figure 6e) [18, 33, 60, 76, 77]. Removing the compression leads to an opposite change in the wrinkle morphology, endowing the flattened bilayer film with high transparency.

To compare the contributions of the changes in amplitude and wavelength to normal transmittance modulation, Meng et al. fabricated two bilayer samples showing different morphological changing behaviors and hence their transmittance modulating sensitivities [76]. They compared the amplitudes and wavelengths of the wrinkles in the two samples when they presented similar opacities (Figure 6f and g). It was found that the wrinkle amplitudes were both close to 0.6 μm, while the wavelengths were 11 and 8 μm. This indicated that the wrinkle amplitude, rather than its wavelength, was the main factor that determined the light refraction, and hence the scattering performance.

Therefore, to improve the transmittance modulating sensitivity to applied strains, the changing rate of the wrinkle amplitude should be increased. According to Eq. (5), in addition to the compressive strain, the wrinkle amplitude is related to the thin film thickness, Young’s modulus, and Poisson’s ratio of the thin film and substrate. Among these, the thin film thickness is the most straightforward parameter to tailor because it is proportional to the wrinkle amplitude and is continuously controllable [18, 76].

By varying the duration of the O_2_ plasma treatment, silica-like SiO_*x*_ thin films with different thicknesses were prepared on the surface of a PDMS substrate (Figure 6h) [76]. Upon stretching, a compressive strain was generated perpendicular to the stretching direction as a result of the high Poisson’s ratio of PDMS, which was employed as the driving force for the formation of surface wrinkles for light refraction. Such wrinkles were considered to be sinusoidal phase gratings diffracting incident light into beams at multiple orders. Wrinkles with a larger amplitude formed under the same compressive strain in a bilayer structure with a thicker SiO_*x*_ film. Under 17% stretching, the wrinkle amplitudes in samples with SiO_*x*_ film thicknesses of 50, 100, 155, and 185 nm were 0.60, 0.95, 1.29, and 1.42 μm, respectively (Figure 6i). The corresponding 0th order diffraction efficiencies of the 635 nm laser were 35, 7.1, 2.4, and 6.5% (Figure 6j), respectively, which resulted in the different transparencies for the four samples under the same strain. In another example, two PVA films with thicknesses of 4.3 and 14.0 μm were prepared by drop-casting on top of PDMS substrates [18]. Correspondingly, highly ordered wrinkles were produced in both bilayer films with amplitudes of 6.9 and 36.2 μm. A similar tendency was observed, where the former sample presented a normal transmittance contrast of only 24%, while it dramatically improved to 70% at the same 10% strain in the latter sample. Because the activation strain for optical modulation is critical to practical applications of mechanoresponsive scatterers, the thickness of the stiff film atop a compliant substrate is a vital parameter to consider when designing the sensitivity to strain.

#### 2.3.3 Crack formation

Thin films fracture into strips upon tension owing to their brittleness. The resultant cracking grooves between the strips have various effects on the final scattering performance, depending on the overall surface structure. For a planar silica/PDMS bilayer film, lateral tensile strains simultaneously generate cracks and wrinkles in the direction perpendicular to the tension [32, 54, 60]. These cracks were generated at mild strains smaller than 10%, lowering the normal transmittance from 92 to 83% [60]. The transmittance drastically decreased to 15% by further stretching as the crack density increased, and wrinkles were formed when the perpendicular compressive strain exceeded the critical strain for buckling. This study suggested that similar to wrinkles, cracks also contributed to the opacity induced by light scattering. To decouple the effects of wrinkles and cracks, a second PDMS layer was used to cover the surface of the SiO_*x*_ layer and prevent wrinkle formation during stretching, forming a PDMS/SiO_x_/PDMS trilayer film (Figure 6k) [76]. Under stretching, cracks evolved in the stiff SiO_*x*_ layer, resulting in a slight optical contrast of less than 20%, even at 50% strain (Figure 6l and 6m). These results demonstrated that cracks have a weaker effect on light scattering compared to wrinkles but can serve as a supplement to increase the transmittance tunability at small strains.

On the other hand, cracks can also be negative to the light scattering effect. As cracks evolve, shear stress accumulates along the film/substrate interface near the cracks. Such stress localization debonds the film from the substrate after cyclic loading; therefore, no wrinkles are observed near the cracks [56]. Moreover, the extensive evolution of cracks allows light to normally transmit through the featureless regions between the wrinkled strips and reduces the opacity [71]. Such cracking phenomena are commonly observed during wrinkle fabrication using a unidirectional prestrain-release process because of the Poisson’s effect-induced perpendicular stretch upon prestrain release [32, 37, 50, 54]. Considering this, intrinsically flexible materials have been chosen for the top thin film to accommodate the large strains that occur during fabrication without the cracking that occurs in brittle materials such as silica [18, 55]. Such a crack-free wrinkle bilayer structure showed a superb transmittance contrast of 85% at a strain of less than 20% [18]. It should be noted that because of the much lower Young’s modulus values of flexible materials, the optimal thin film thickness can be increased beyond 10 μm, which is much higher than the nanometer-scale thickness when conventional brittle materials are used. Another method to hinder crack generation is to modify the fabrication process. In contrast to its unidirectional counterpart, bidirectional compressive strain generates stresses of equal magnitude along the two orthogonal axes, which hinder the brittle thin film from cracking [71]. These bidirectional prestrains should be released simultaneously; otherwise, perpendicular tension will still be induced [51]. The normal transmittance of an as-fabricated scatterer with 2D buckles increased from ∼10% in the buckled state to ∼80% in the flattened state. If the applied tensile strain exceeded the prestrain, cracks were generated, giving rise to an even higher transmittance. This crack-related transmittance increase attested to the detrimental effect of cracks on the opacity when extensive cracks develop.

#### 2.3.4 Wrinkle orientation

Wrinkles are generated by compressive strains, so they orient along the compression axis. The orientation of the wrinkles determines the orientation of the light diffraction patterns and hence the directional light scattering behavior of the scatterer [77], [78], [79], [80], [81]. To investigate the relationship between the diffraction pattern orientation and the wrinkle morphology, the diffraction patterns of the white light passing through a 1D wrinkled silica/PDMS specimen re-stretched along the prestrain direction were studied (Figure 6n) [56]. Initially, when no strain was applied, the white light was diffracted and appeared blue at the center and red at the end, which was oriented along the prestrain direction (Figure 6o). Interestingly, when a strain close to the prestrain was applied, the diffraction pattern became orthogonal; its orientation was parallel and perpendicular to the prestrain direction. This was attributed to the coexistence of ordered structures in both orthogonal directions. Because of the Poisson’s ratio of PDMS, a secondary wrinkle was produced by the perpendicular compressive strain as the first wrinkle was flattened by the applied strain. As long as the initial prestrain was twice larger than the perpendicular compressive strain, the two orthogonal wrinkles could coexist. The diffraction induced by the first wrinkle disappeared, while that perpendicular to the prestrain became more predominant as the applied strain increased to a sufficiently large value to flatten the first wrinkle. Wrinkle-orientation-dependent directional light scattering was also reported by Wu et al. [82]. It was demonstrated that a circular laser beam was diffused isotropically by 2D labyrinth wrinkles so that a heart-shaped pattern was visible regardless of the light source/observation angle (Figure 6p). However, the pattern in the sample with 1D wrinkles was visible at 90°, but not at 0°. Such an anisotropic light scattering behavior could be utilized for anti-peep displays (Figure 6q).

## 3 Design of 3D scatterers for effective light modulation

This section summarizes very recent developments of 3D scatterers with the perspective of their design concept, underlying physics, and demonstrated mechanical and optical features that can be further expanded to multifunctionality.

### 3.1 Formation of light scattering sites inside transparent medium

**Figure 7:**
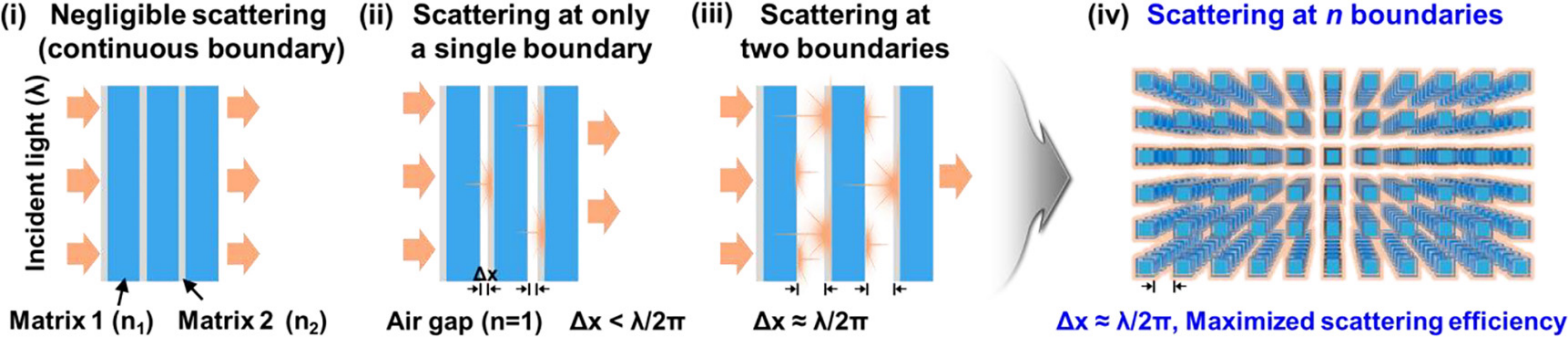
Schematic illustrations showing engineering strategy for controlling scattering based on dependence of light scattering effect on distance between neighboring boundaries. Reprinted with permission from Ref. [17].

An interfacial engineering strategy for controlling the gap between two different neighboring materials (*e.g.*, particles, pores, or grain boundaries) in an optically transparent medium [17], especially in a case where χ≈1 , which refers to Mie scattering, offers the capability of scattering broadband visible light in a forward direction with relatively larger intensity than those in the reverse direction (Figure 7). For example, when the binary phases are conformally contacting each other, only negligible reflection at the interfaces occurs as a result of the formation of continuous boundaries (Figure 7i). When its size increases but is still smaller than λ/2π , the gap serves as a single boundary where relatively enhanced scattering occurs (Figure 7-ii). As the gap grows beyond λ/2π , it offers a pair of air/phase boundaries that behave like autonomous boundaries for light scattering, thereby facilitating significantly enhanced scattering compared to other cases (Figure 7-iii). Interestingly, with these attributes, it is easy to understand that the total scattering efficiency can be maximized when the light travels through more complicated 3D scattering phases with multiple boundaries (Figure 7-iv). If it was possible to simultaneously manipulate the 3D heterogeneous interfaces in a composite, such a strategy would be an effective way to adjust the light transmission for high-contrast optical modulation.

#### 3.1.1 Dispersed nanoparticles in elastomeric matrix

Various classes of 3D porous structures (*i.e.*, paper, assembled powders, and mesh) commonly exhibit complete opaqueness because the incident light across the entire visible spectrum actively scatters at the multiple scattering layers in the 3D structures [3]. It is expected that strain-induced optical modulation could be significantly improved, compared to cases based on 2D scatterers, if this optical maze-like structure could be used to control the light scattering effect through structural/interfacial modifications.

**Figure 8:**
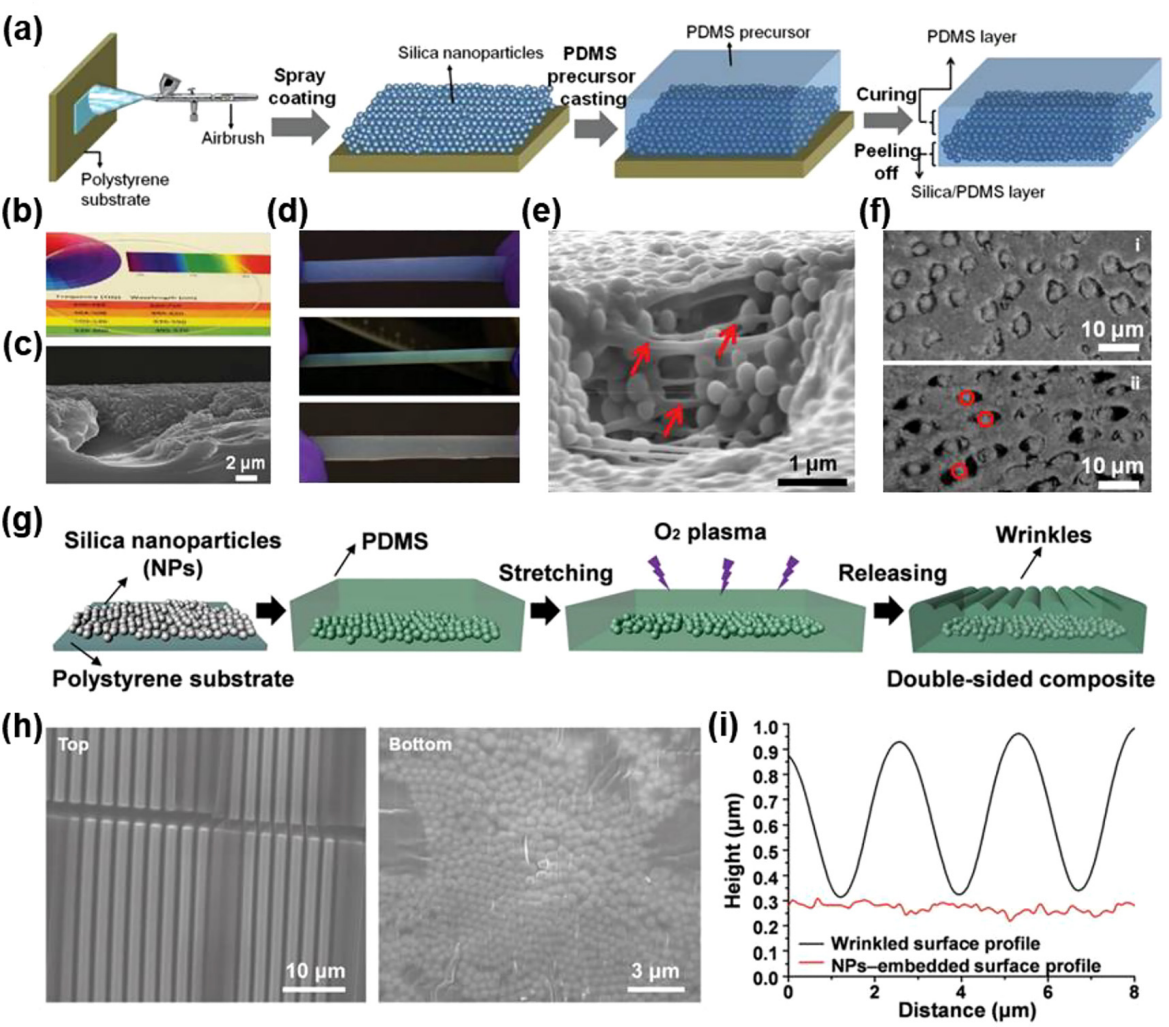
3D hybrid nanocomposite based on random network of self-assembled nanoparticles for mechanoresponsive scatterer. (a) Schematic illustration of the fabrication process. (b) Digital image and (c) SEM images of the transparent silica/PDMS composite film (unstretched). (d) Digital photographs of stretched films with embedded nanoparticles of various diameters: i) 221, ii) 258, and iii) 306 nm. (e) SEM image of the stretched silica/PDMS film with 258 nm diameter nanoparticles at 80% strain. (f) Optical images of PDMS film with i) unstretched and ii) stretched silica microparticles (diameter of 5 µm). The circles indicate silica nanoparticles, and the black regions indicate the voids. (g) Fabrication of wrinkle–silica particle composite film. (h) Top-view SEM images of the top and bottom surfaces of the composite in the released state. (i) AFM scan profile of the composite film at released state. Reprinted with permission from Refs. [83, 84].

A simple but effective strategy for optical tunability by introducing a hybrid nanocomposite consisting of silica nanoparticles (NPs) dispersed in a PDMS matrix was developed by the Shu Yang’s group [83, 84]. Silica NPs have a refractive index (*n*) of 1.47 with PDMS (*n* = 1.43), enabling the high optical transparency of the as-prepared hybrid nanocomposite before stretching [83, 85]. Upon stretching, however, the interval between the assembled NPs changed and/or air gaps were formed because of the different mechanical responses of the ceramic and elastomer to the applied strain. Therefore, the color and/or optical transparency could be reversibly changed in response to mechanical deformation. Specifically, as shown in Figure 8, silica NPs were spray-coated on a flat substrate and then peeled off after infiltrating the PDMS prepolymers and the subsequent curing process for the PDMS (Figure 8a). The resultant silica NP/PDMS composite exhibited a transparency with a transmittance of more than 90% in the visible spectrum, as a result of the refractive index matching of the system (Figure 8b and c). Because of the quasi-periodicity of the self-assembled silica NPs, angle-independent structural color that could be finely controlled by the size of the silica NPs was observed at a strain of more than 40% (Figure 8d). More importantly, the simultaneous formation of a strain-induced air gap (*n* = 1.00) occurred at the interface of the silica and PDMS, leading to a significant transmittance drop of 30% as a result of the active light scattering (Figure 8e and f). Further, the optical response sensitivity could be further improved by reducing the activation strain level to achieve a meaningful level of transmittance modulation (>50%), which showed its great potential for smart window applications with a fast response, high optical modulation performances under large deformation, and structural coloration. Kim et al. suggested a modified composite design that combined a 3D hybrid nanocomposite and wrinkles to improve the contrast of optical modulation at a smaller strain level (<25%) [84]. Silica NPs were embedded at the bottom of the composite film, while wrinkles were fabricated on the top by treating the pre-strained PDMS (<20%) with oxygen plasma (Figure 8g and h). The synergistic effects of the co-existing 2D scatterer based on surface wrinkles and 3D scatterer based on embedded NPs resulted in substantial changes in the optical transmittance with a relatively small strain of 10%. The multistate smart windows showed reversible optical switching performances ranging from the highest transmittance of 86.4% at 10% strain (the pre-strain level) to the lowest transmittance of 25.2% at 550 nm, with an additional 30% strain to the pre-strain level (for a total of 40% strain). Based on the simple fabrication procedures and the material composition of the hybrid nanocomposite, these multistate smart windows revealed potential suitability for vehicles, indoor windows (*i.e.*, an office), and outdoor windows (*i.e.*, a building shell), which could demonstrate both a transparent state and dynamic optical modulation.

#### 3.1.2 Highly periodic 3D nanonetwork for 3D scatterers

**Figure 9:**
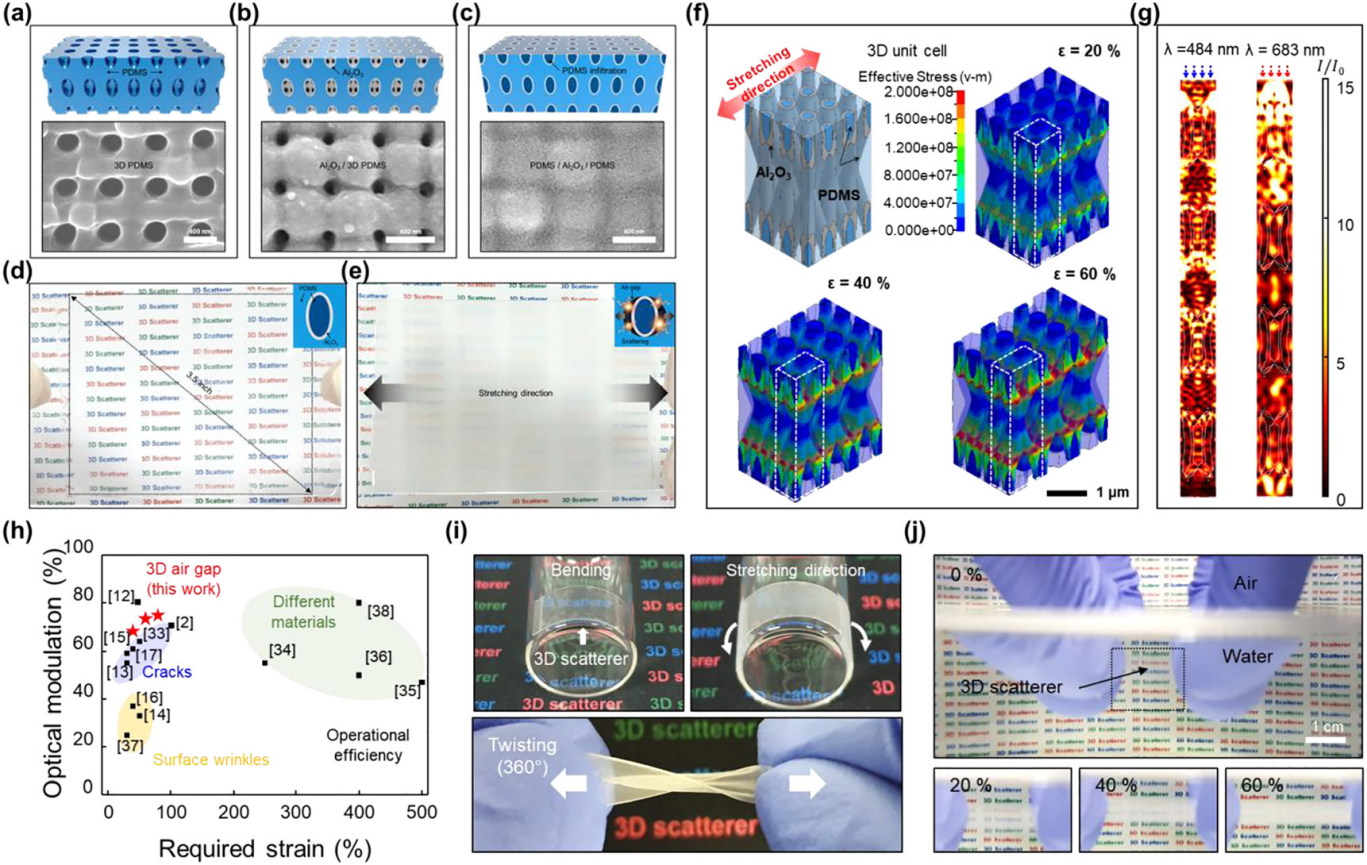
3D scatterer with 3D heterogeneous interfaces in highly periodic 3D nanocomposite. (a) Fabrication of 3D PDMS, (b) the Al_2_O_3_-nanoshell-coated 3D PDMS, and (c) the hybrid nanocomposite infiltrated with PDMS. (d) Digital images of 3D scatterer before (transparent) and (e) after stretching (opaque). (f) Stress distribution in nine unit cells of a 3D scatterer with an Al_2_O_3_ shell thickness of 60 nm under strains of 20, 40, and 60%. (g) Electromagnetic field intensity distribution for 484 and 683 nm light passing through the 3D scatterer. (h) Comparison of the reported optical modulation performances with different types of the scatterers. (i) Robustness of a 3D scatterer under dynamic mechanical deformation and (j) water-immersion conditions. Reprinted with permission from Ref. [17].

Although particle-embedded PDMS composites have shown effective optical modulating performances under mechanical stimuli, an in-depth study of the mechanism should be conducted by investigating the structure/property correlation to further improve the practical use of such scatterers. Recently, based on expertise in the fabrication of many classes of 3D nanocomposites and their applications [17, 86], [87], [88], [89], [90], [91], [92], [93], [94], [95], [96], our group proposed a novel design concept for a 3D scatterer based on a highly periodic network structure that allows systematic control of the optical and mechanical properties to define key considerations and reveal optimized layouts [17]. Specifically, by incorporating an ultrathin Al_2_O_3_ nanoshell structure (with a thickness of ∼60 nm) in the elastomer (PDMS), a thin (∼13 µm) but optically dense 3D scatterer was fabricated to control the 3D heterogeneous interfaces (Figure 9a–c). The contrast of the optical modulation increased with the total thickness of the 3D structures [97]. However, to simultaneously achieve film stretchability, conformability, and, more importantly, initial transparency, the material system should be carefully optimized to achieve a high optical density within the thin film thickness [17, 83].

The as-prepared hybrid composite was transparent (90% initial transmission) because of index matching between the Al_2_O_3_ (*n* = 1.76) and PDMS (*n* = 1.43) (Figure 9d), but upon stretching, strain-induced air gaps simultaneously occurred in the composite, leading to a drastic reduction in the transmittance to 16% (Figure 9e). The underlying physics, which involved light scattering at the heterogeneous interfaces of the air gaps, was verified by building 3D unit cell models. As shown in Figure 9f, air gaps were continuously generated on both sides of the center ellipsoid and the bridge elements of each 3D unit cell because of the forced shaping of the high-modulus oxide in the elastomer. As the applied strain increased to 60%, the volume fractions of the air gaps increased to 37.5 vol%. Indeed, this growing tendency of the air gap induced active visible light scattering. Based on the light scattering theory [19, 20], the effective size of an air gap capable of scattering light was calculated to be approximately 60 nm. When the gap size was larger than 120 nm, the scattering efficiency became saturated because the scattering interfaces were fixed to have only a pair of index-mismatched boundaries separated by air gaps. To confirm the contributions of the air gap as a scatterer to the transmittance drop, a multi-physical analysis was performed. Over the cross-section of the center unit cell model of the 3D scatterer, the significant enhancement of the electromagnetic intensity at the heterogeneous interfaces was direct evidence of active light scattering (Figure 9g). As exemplified by both short wavelength (484 nm) and long wavelength (683 nm) incident light, the uniform light scattering through the entire 3D structure suggested that the 3D scatterer could serve as a rational structural platform for high-contrast optical modulation. Therefore, the systematic analysis presented a scatterer with a highly periodic network that assumed roles that were different from those of random structures in terms of further structural diversity and uniformity, which would expand its scalability.

For the practical application of this scatterer, it is desirable to reduce the required strain level (<20%) to activate the high optical switching performance, which is plotted in the upper left part of the strain versus the tunable transmittance (specular transmittance change Δ*T* = *T*_initial_ − *T*_stretched_) plot (Figure 9h). Because of the enhanced optical density of the 3D scatterer compared to a 2D scatterer with the same film thickness, a very large transmission modulation (Δ*T*) of up to 74% was achieved at visible wavelengths under 60% tensile strain, which greatly exceeded the results of 2D scatterers. As shown in Figure 9i, the operations were not limited to the stretching deformation; they could also include bending and twisting through 360° owing to the superb flexibility of the 3D scatterer. More importantly, the stretchable film was successfully demonstrated to maintain its performance even in water. Because the scattering sites (air gap, *n* = 1.00) embedded in the PDMS matrix (*n* = 1.43) were protected from the refractive index matching with the surrounding water medium (*n* = 1.33), the scatterer did not lose its functionality as a result of interference from the neighboring water (Figure 9j). Therefore, the design concept for this 3D scatterer offers new insights into the application of a variety of high-value-added utilizations such as privacy protection, smart windows, and biological applications.

### 3.2 3D mechanical heterogeneity for mechanochromism

The aforementioned 3D scatterer exploits multiple scattering inside a periodic 3D structure. Despite the high contrast of the scatterer, its initial transmittance above 90% was sacrificed because of the Al_2_O_3_ intermediate layer, which had a high refractive index (*n* = 1.76). In addition, a strain greater than 60% was necessary for contrasts beyond 50%. The initial transparency and efficient formation of air gaps are two critical factors to consider to further improve the modulation range and sensitivity. Structure optimization could be the key to improving the mechanochromic sensitivity without sacrificing the initial transmittance. Chen et al. recently proposed the introduction of mechanical heterogeneity into 3D structures. The mechanochromic sensitivity was expected to improve because there is a strain threshold for generating air gaps with efficient sizes for Mie scattering [89].

The resultant 3D heterogeneous nanocomposite consisted of two interdigitated PDMS phases with different Young’s moduli and a silica intermediate layer. The silica layer was generated by a simple UVO plasma treatment, which weakened the interface strength between the two PDMS phases to below 200 kPa. Silica has a refractive index (1.46) close to that of the PDMS matrix (1.43), thus endowing the scatterer with a high initial transparency of 93%. The modulus of the PDMS was controlled by the base-to-curing-agent mixing ratio, with a higher mixing ratio giving rise to a denser crosslinking network and higher PDMS stiffness. The mixing ratio and Young’s modulus of the soft PDMS (s-PDMS) were fixed at 15:1 and ∼1.1 MPa, while the ratio for the hard PDMS (h-PDMS) was tuned from 15:1 to 1:1, corresponding to Young’s modulus values of 1.1–4.2 MPa. As such, the nanocomposite was homogeneous when the h-PDMS ratio was the same as that of the s-PDMS and most heterogeneous when the ratio was 1:1.

**Figure 10:**
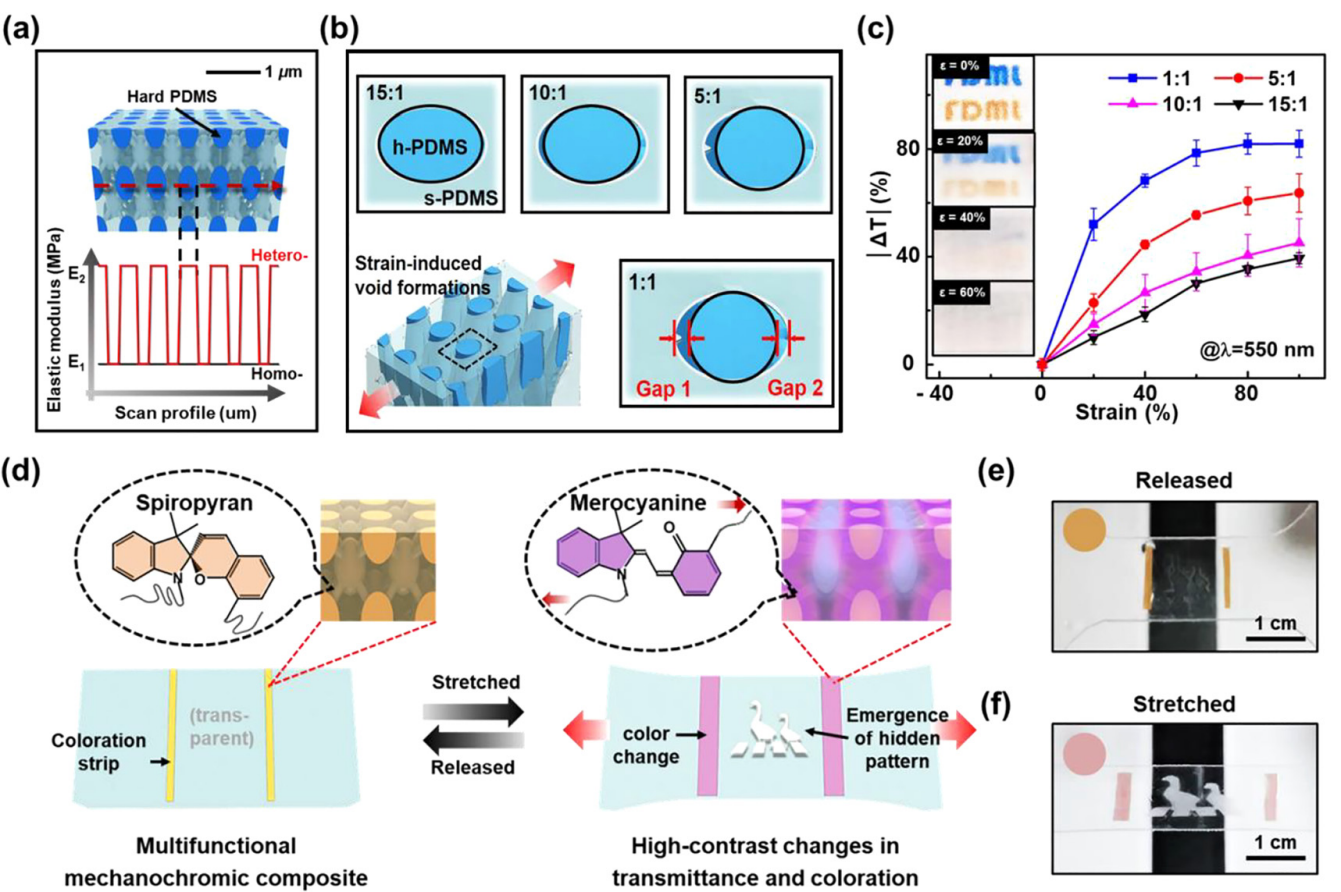
Mechanical modification of 3D scatterer to improve optical modulation performances. (a) Modulus mismatch in the interdigitated 3D network. (b) Nanogap formation at 30% strain in 3D models with different heterogeneity values in FEA simulation. (c) Transmittance modulating performance of 3D heterogeneous scatterers as a function of modulus mismatch and applied strain. (d) Schematic illustrations showing the simultaneous modulation of transmittance and color by preparing multifunctional mechanochromic composites and the resulting images under (e) released and (f) stretched states. Reprinted with permission from Ref. [89].

As illustrated in Figure 10a, the modulus changed regularly along the scanning line in the heterogeneous nanocomposite, which induced periodic stress/strain localization at the modulus mismatched interfaces. A finite element analysis indicated that compared to its homogeneous counterpart, the heterogeneous nanocomposite exhibited over 50% higher strain localized at the interface between the h- and s-PDMS phases. The localized strains caused the two phases to debond at a lower applied strain, thus greatly facilitating the air gap formation process. As shown in Figure 10b, gaps were formed on both sides of the h-PDMS core along the stretching direction. It was found that when the same strain was applied, significantly larger gaps were generated in the heterogeneous composite, while those in the homogeneous composite were negligible. Such an effect was magnified in the interdigitated 3D structure, making the nanocomposite highly sensitive when used as a mechanoresponsive scatterer.

The heterogeneity effect on light scattering was proved experimentally by comparing the mechano-optical behaviors of the scatterers with different extents of modulus mismatch (Figure 10c). Both the modulation range and sensitivity improved dramatically. The scatterer 1:1 presented an extraordinary contrast of 82%, while the maximum contrast for the scatterer 15:1 was only 40%. The sensitivity was enhanced as the heterogeneity in the 3D structure increased, and a decent contrast of 50% could be activated by applying a strain as low as 15% to the scatterer 1:1. Owing to the efficient formation of air gaps, optically dense materials in the intermediate layer became unnecessary for enhanced light scattering. Compared to the scatterer with the Al_2_O_3_ intermediate layer, the scatterer consisting of PDMS/silica/PDMS eliminated the slight scattering at the PDMS/Al_2_O_3_ interface and presented a higher initial transparency due to the refractive index matching between the compositions. Moreover, the expensive and hour-long atomic layer deposition (ALD) process was replaced by a simple UVO treatment that is within 10 min, making the fabrication processes more practical and approachable.

This 3D heterogeneous strategy has been proven to be broadly applicable to the design of materials with different mechanochromic properties. In addition to facilitating air gap generation for transmittance modulation, strain localization could be exploited to enhance coloration contrasts in mechanophore-dyed nanocomposites when two phases with different moduli are bound without separation. Based on the 3D heterogeneous structure, a hybrid mechanochromic membrane integrating nanocomposites with weak and strong interfaces for light scattering and coloration, respectively, was demonstrated (Figure 10d). The multifunctional membrane was transparent in the middle, with two orange stripes on the sides upon release (Figure 10e). Interestingly, when stretched, the membrane presented vivid simultaneous changes in its transmittance and color (Figure 10f). The geese pattern encrypted in the center was disclosed as light passing through the patterned region became scattered. Meanwhile, the mechanophore in the coloration strips was activated, bringing about a strong color change from orange to purple. In short, the 3D heterogeneity concept that utilizes the stress/strain concentration was proven to be a powerful tool for designing various mechanochromic systems.

### 3.3 Large-area production of 3D scatterer and its multifunctionality

The demand for a scalable scatterer beyond the wafer dimension that also exhibits superb uniformity over the entire active area and its reliable operation is growing in many large-scale application areas [8, 30]. However, the widespread adoption of scatterers for these purposes is technically challenging because of the absence of a reliable fabrication tool for mass production.

**Figure 11:**
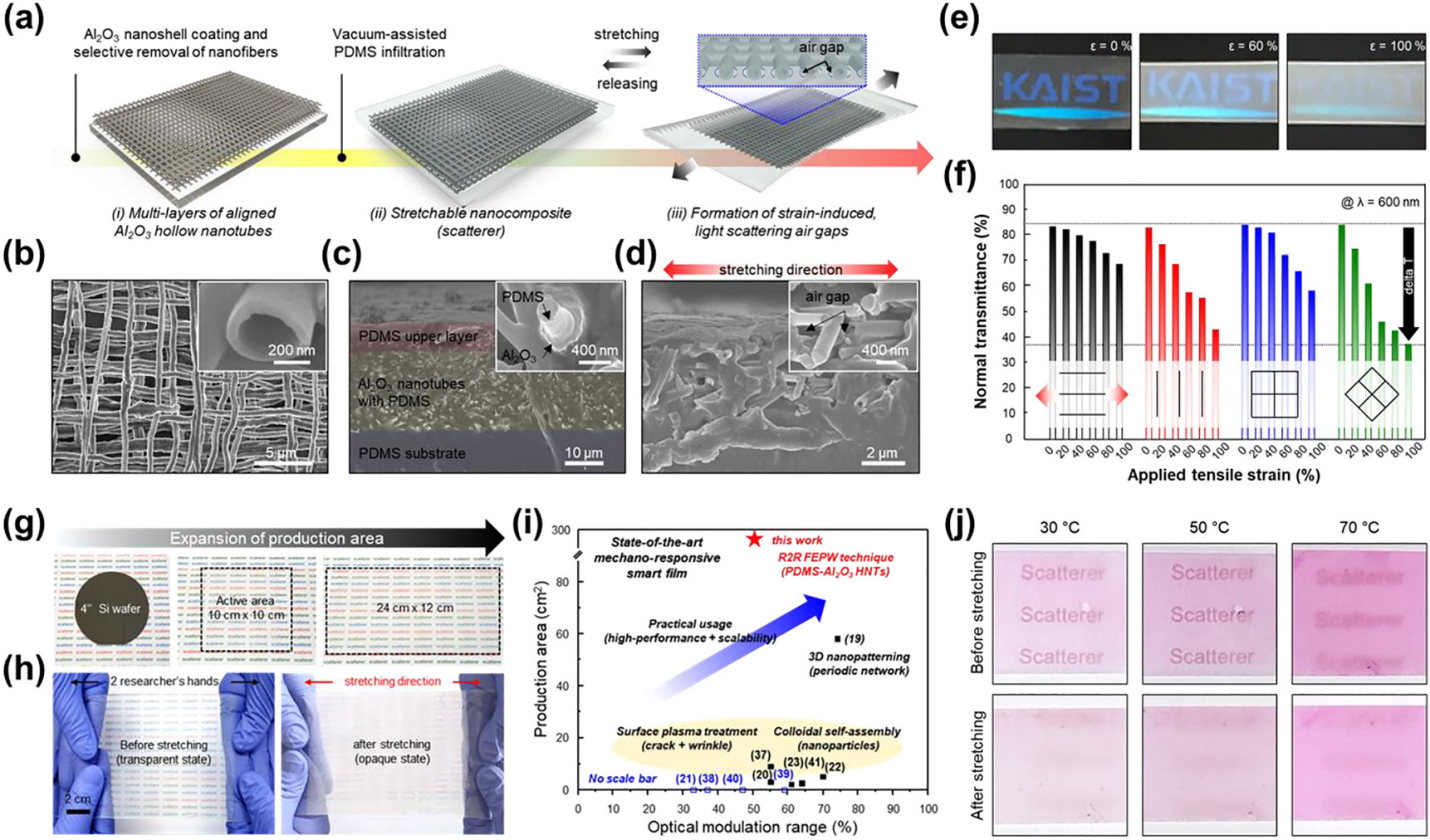
Design of scatterer based on aligned nanofiber structures. (a) Schematic illustrations showing the fabrication procedures for the scatterer. (b) Top-view SEM image of Al_2_O_3_ hollow nanotubes on a PDMS substrate. (c) A cross-sectional view of the nanocomposite infiltrated with PDMS. (d) Strain-induced air gap formation in the composite. (e) Digital images of a scatterer with a rhombus-like structure and (f) normal transmittance values of scatterers with various morphologies such as parallel, vertical line structure, check pattern, and rhombus-like structure. (g) A large-area scatterer before and (h) after stretching. (i) Comparison of the previously reported production areas of scatterers. (j) The thermal-dependent color change in a scatterer designed by incorporating a thermochromic dye into the composite. Reprinted with permission from Ref. [34].

This section highlights a recent strategy that uses the direct polymer writing technique to provide a straightforward design opportunity for creating large-area 3D architectures with customized geometries [34]. Cho et al. developed a multi-stimuli-responsive scatterer designed for an ultra-large area (near 300 cm^2^) *via* focused electric-field driven polymer writing (FEPW). As a modified electromechanical system based on the conventional electrospinning technique, the electrospun polymeric nanofibers (NFs) jet from the nozzle tip and are guided linearly and perpendicularly by simply placing two insulating blocks in the middle of the path between the nozzle tip and the target collecting substrate. The geometry of the NFs (fiber thickness and dimension) and the assembled 3D structure (parallel, vertical, check-patterned, and rhombus-like pattern) could be elaborately designed by dynamically controlling the forward/backward motion, as well as the rotation of the target substrate at a constant speed (5 mm/s). Figure 11a shows the overall fabrication procedure for the 3D scatterer starting from the preparation of a water-removable template with poly(ethylene oxide) (PEO). The assembled 3D PEO membrane structure served as the scaffold for coating ultrathin (∼60 nm) layers of Al_2_O_3_ using the ALD technique. Then, periodic alumina nanotubes were replicated from the 3D NF membrane by selectively dissolving PEO in water (Figure 11b). The vacuum-assisted infiltration method allowed the 3D nanotube structure to be inserted into the PDMS without any voids (Figure 11c). After PDMS curing, the stretchable hybrid composite provided a large number of heterogeneous interfaces upon stretching, inducing active light scattering sites in the form of air gaps parallel to the stretching direction (Figure 11d).

Because of the matching of the effective refractive index, the as-prepared composite films with aligned structures showed high optical transparency (Figure 11e). However, once the applied tensile strain was increased to 100%, the film continuously turned into an opaque state because of the significant light scattering at the heterogeneous interfaces. To investigate the critical role of geometric factors in the quality of the optical modulations, the morphologies of the 3D nanopatterns were controlled to form four aligned cases (*e.g.*, parallel line, vertical line, check-patterned, and rhombus-like structures), comparing with the randomly distributed case. The FEPW writing time and Al_2_O_3_ nanoshell thickness were fixed at 12 h and 60 nm, respectively. Because of the presence of structural components oriented parallel to the stretching direction in the parallel and check-patterned cases, a low optical modulation performance (Δ*T*) of up to 25% was achieved (Figure 11f). The isostrain condition hindered effective air-gap formation, even under large mechanical deformation. On the other hand, the vertical and rhombus-like cases were found to be relatively free from such limitations. Because of the high volume of in-plane scattering sites in the travel direction of the light, among the controlled morphologies, the rhombus-like case showed the highest transmittance drops until saturation at a transmittance of less than 40% from the transmittance of nearly 90% at visible wavelengths (Figure 11f). The optical tendency linearly followed the applied strain levels, indicating the capability to quantify the mechanical deformation level.

Equally important was the realization of a scalable production scheme to utilize the 3D scatterer for distinct applications such as climate-adaptive building shells and esthetic optical diffusers. By combining the FEPW, a roll-to-roll (R2R) system with multiple nozzles (>4), and a continuously movable R2R collector, the production area for the aligned membranes of the scatterer could be successfully expanded to 1750 cm^2^. It should be noted that the folding of the membrane during the ALD process could solve the spatial limitations imposed by the physical dimensions of conventional laboratory-scale ALD equipment (<8 in). Owing to the interconnected open porosity (>90%) of the membrane, it was possible to obtain Al_2_O_3_-coated membranes with sizes of 10 × 10 cm and 24 × 12 cm (Figure 11g). Thus, a large-area scatterer was realized (Figure 11h), and more importantly, the achieved production scale greatly exceeded the reported size of a scatterer membrane to date (Figure 11i).

The technical features of this solution-based polymer writing process included the incorporation of various functional materials on the surface of polymeric NFs as a one-pot fabrication method. In the FEPW step, a thermochromic dye (polyoxymethylene melamine) was added, followed by the fabrication of the scatterer. The resulting multifunctional (mechano- and thermochromic) membrane simultaneously showed a color change from powder pink to deep pink with an increase in temperature from 30 to 70 °C and a positive effect on the applied mechanical strain (Figure 11j). By extracting the RGB value, the strain and thermal-induced optical changes made it possible to quantify the associated stimuli levels.

## 4 Other strategies for mechanically induced optical modulations

In addition to the above-mentioned 2D scatterers showing surface feature changes and 3D scatterers utilizing internal delamination, there are other scatterers driven by mechanical stimuli that are based on other strategies and working mechanisms.

### 4.1 Optical shutter

Window shutters or blinds are the most common commercial window dressing products that are used to adjust the amount of incoming sunlight. Light transmission through windows is high when the slats on the shutters are parallel to the incident direction but lower once the slats rotate in the transverse direction. Inspired by window shutters, researchers have developed various smart optical materials consisting of rotatable micro-/nano-components for adjustable light scattering, reflection, and absorption [98], [99], [100], [101].

**Figure 12:**
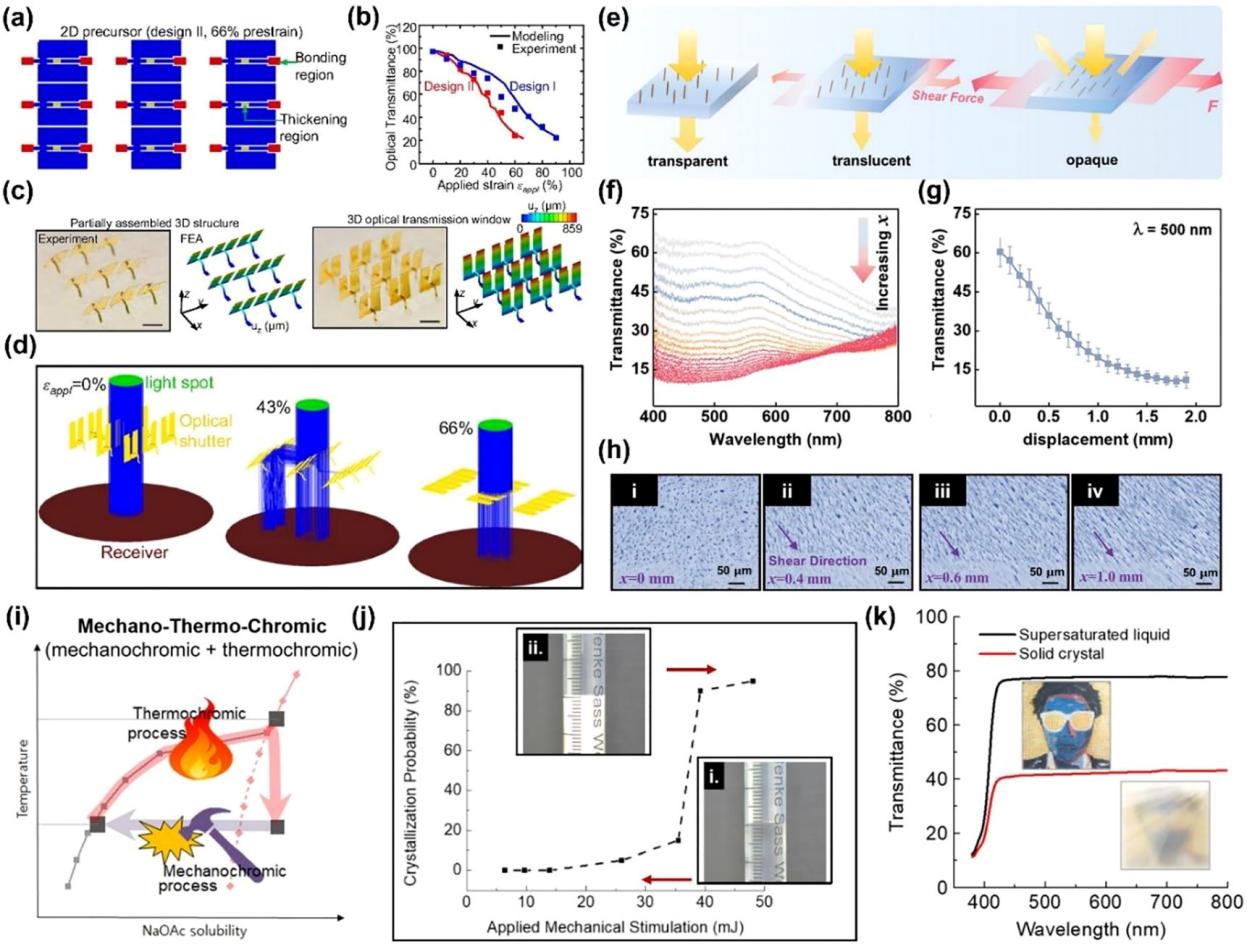
Other strategies to design mechanoresponsive scatterers. (a) Schematic illustration of an optical shutter and its regions of bonding (red rectangles). (b) Measured and calculated optical transmittance as a function of uniaxial strain applied to an elastomeric substrate. (c) Modeling predictions of deformations at the intermediate state (left) and final state (right, scale bars, 500 μm). (d) Illustrations of the simulated light paths for devices with engineered thickness variations, at three different levels of stretching. (e) Schematic illustration of a shear-responsive smart window at different states: transparent state with vertically arranged chains, translucent state with slightly tilted chains under a small shear force, and opaque state under an increasing shear force. (f) Transmittance spectra of the SR SW under different shear displacements. (g) Dependence of transmittance on shear displacements at *λ* = 500 nm. (h) Top-view optical microscopy images of nanochains in a PAM-NCs hydrogel with different shear displacements. (i) Solubility graph of different temperatures for sodium acetate anhydrous (CH_3_COONa, red line) and sodium acetate trihydrate (CH_3_COONa·3H_2_O, purple line). (j) Crystallization probability of sodium acetate with the applied mechanical stimuli. Insets: digital images of sodium acetate before and after crystallization. (k) Spectral transmittance of a mechano-thermo-chromic device with a supersaturated liquid phase (black line) and crystallized solid phase (red line). Reprinted with permission from Refs. [98, 99, 108].

Zhang et al. proposed a reflective membrane bonded to a transparent, uniaxially prestrained PDMS substrate through its two ends (Figure 12a) [99]. Because of the two Kirigami cuts on the membrane, compressive buckling occurred in the central ribbon when the prestrain was released, forcing the membrane to rotate upward and orient perpendicularly to the substrate. Because the intrinsic light absorption by the materials was minimized, the optical transmittance of the 3D mesostructure in the buckling state was as high as 97% (Figure 12b), surpassing the maximum transmittance in most reported mechanochromic materials [8]. On the other hand, the light was blocked by the reflective membrane as a consequence of the flattening of buckles by the applied strains (Figure 12c and d). A considerable transmittance modulation of 75% was achieved with an activation strain of 66%. Similarly, the tilting orientation of the cut units in a Kirigami structure upon deformation has been harnessed to diffuse excessive solar radiance for energy saving [100, 101]. The key to the high sensitivity of these optical shutters based on metamaterials lies in increasing the rotation angle at a given strain. For example, to enhance the rotation of the 3D mesostructure, the thickness of the ribbons in the center was increased from 8 to 23 μm [99]. The original structure and the modified structure with a larger thickness are referred to as Design I and Design II, respectively. As shown in Figure 12b, Design II required a lower strain of 66% for the full-range optical modulation compared to that of 90% for Design I because of the reduced curvature in the thickened ribbons.

Recently, the same principle was applied by Li et al. to develop nanocomposites composed of rotatable Fe_3_O_4_ nanochains (NC) for smart window applications, with a much lower fabrication cost than conventional photolithography [98]. Vertically aligned Fe_3_O_4_ NCs were first prepared by orienting NCs in a magnetic field. Then, the transparent hydrogel matrix was cured to anchor down the alignment. The orientation of the NCs under shear caused the smart window to change from transparent to translucent and opaque, with more incident light being reflected and scattered by the increased projected area of the tilted NCs in the vertical direction. The transmittance of the nanocomposite gradually decreased from 65 to 10% as the shear strain increased (Figure 12f and g). Although the shear strain was 50% when the shear displacement was 1.5 mm, the “strain” defined as the ratio of the shear displacement to the original lateral size along the strain direction was only 5%, which was significantly smaller than the values for the majority of scatterers that are driven by lateral tensile strain [54, 55, 62, 69]. The light-tuning mechanism of the rotating NCs was verified using optical microscope images, where the scattered dots became short lines along the shear direction (Figure 12h). In contrast to optical modulating devices based on 3D mesostructures, the key to the high sensitivity of an NC-based nanocomposite is the concentration of the nanochains. Although the optical modulation range was found to improve with an increase in the NC concentration, both low and high concentrations falling outside of the range suppressed the modulation range.

### 4.2 Phase change materials

While phase change materials (PCMs) have been extensively employed for developing electrochromic [29, 102], [103], [104] and thermochromic devices [11, 105, 106], their use in mechanochromic devices remains largely unexplored [107, 108]. Among the PCMs, sodium acetate (NaOAc) is a widely used water-soluble salt that can form hydrates with water molecules [109]. As a saturated sodium acetate solution becomes supersaturated when the temperature decreases, sodium acetate spontaneously crystalizes into sodium acetate trihydrate when there is an external mechanical stimulus, turning the transparent solution into a murky dispersion (Figure 12i). This reaction can be reversed by applying heat.

Based on this phenomenon, Cho et al. first proposed a novel chromatic device that was responsive to mechanical and thermal stimuli [108]. The device was simply composed of a supersaturated sodium acetate solution sealed in a PDMS/glass chamber that was integrated with a Joule heater. Because the solubility of sodium acetate at 65 °C is 140 g/100 g H_2_O, which is much higher than the 50 g/100 g H_2_O value at 50 °C, almost all the NaOAc crystallized when mechanical stimulation was applied (Figure 12j), endowing the solution with a milky opaqueness. The transmittance dropped from ∼80 to 40% from the supersaturated state to the crystallized state as an external mechanical stimulus was applied, making the portrait behind invisible (Figure 12k). While the composition and fabrication were simple, the exhibited transmittance contrast was only 40%, and the minute-scale response time was much longer than that of conventional mechanoresponsive scatterers (within a couple of seconds). Moreover, the crystallization could be activated by mechanical disturbances. Such a nonspecific requirement for an activation stimulus means that the transparent state is not stable, which greatly limits the practical application of this new type of scatterer.

In addition, crack opening on light-absorbing/reflecting membranes [110, 111] has been demonstrated for transmittance modulation. The applied strains fractured the light-absorbing carbon nanotube (CNT) film, changing the black CNT/Ecoflex composite translucent. The laser light intensity after passing through the film could indicate the applied strain, making the film a strain sensor showing optical responses [110]. In another report, an adaptive fluid-infused porous film was proposed with a transparency and wettability that could be tuned by the tensile strain. The film was originally transparent as the pores in the structure were filled with a liquid with a refractive index matching that in the elastomer matrix, but then turned opaque. However, when the pores expanded under a strain, the fluid retreated into the pores, exposing the rough feature of refracted light [112].

## 5 Applications

With the ultimate goal of reducing the global energy consumption and simultaneously producing a positive impact on indoor environmental quality, over the last decade, smart window technology has experienced rapid development driven by growing worldwide interest. Indeed, novel classes of windows designed to be activated by external stimuli (*e.g.*, electro-, thermo-, and photoresponse) have been created [8, 30]. Examples can be found in smart windows installed in buildings, automobiles, and displays. Even more interestingly, commercialized models of electrochromic and thermochromic devices are now available on the market. Despite their active optical modulations, there are still some challenges to address, including price competitiveness, mass production, the external power supply needed to retain the controlled state, and their complicated fabrication processes [8]. In contrast, as described in the previous sections, the scattering configuration has emerged as a scheme that provides a straightforward operation, cost-effectiveness, and outstanding levels of optical modulation with instant transitions, compared to electro- and thermal activations [17, 34]. This section reviews the recent demonstrations of mechanoresponsive scatterers.

**Figure 13:**
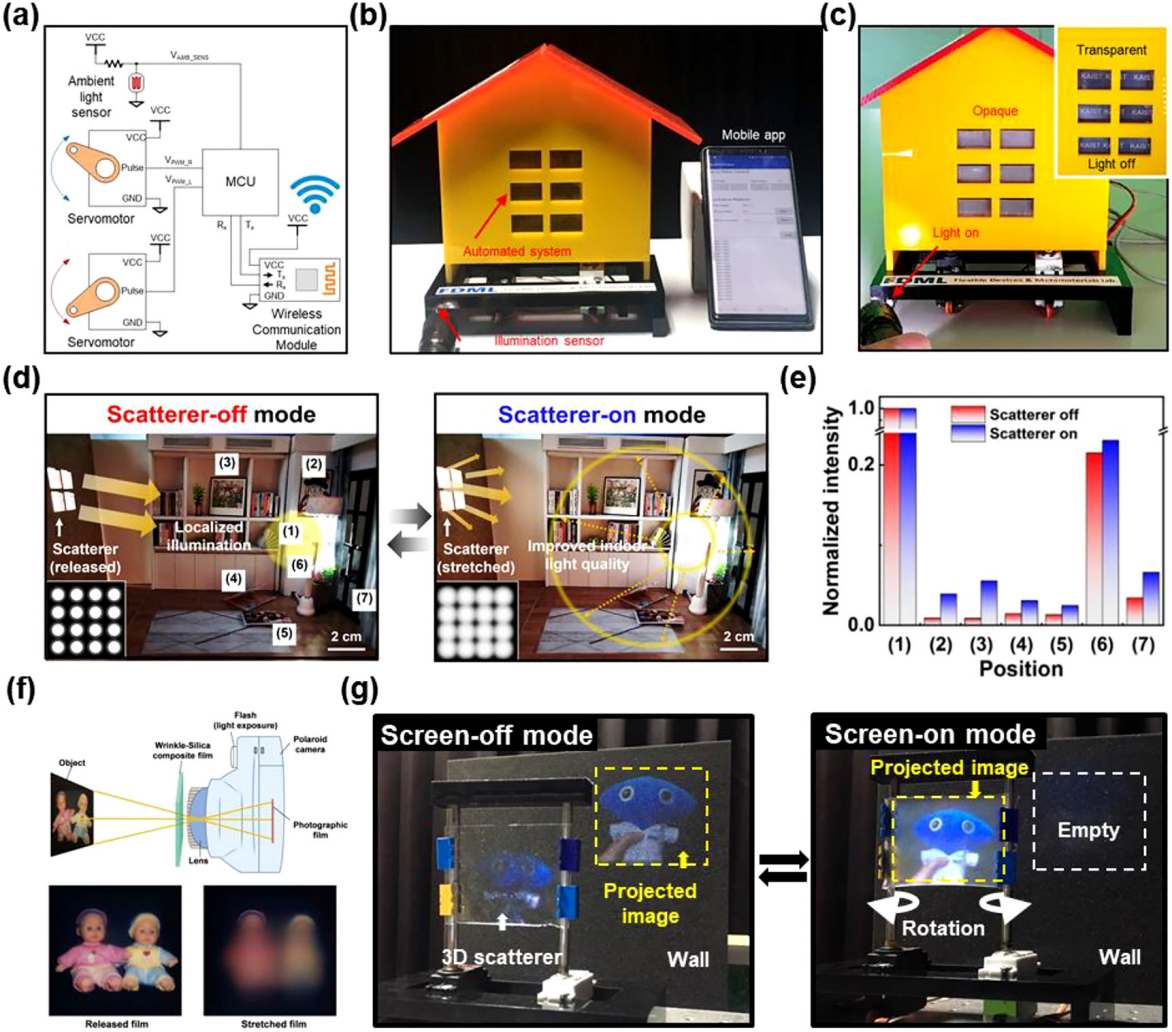
Emerging applications of scatterers using smart optical modulations. (a) A circuit diagram and (b) an overview of a self-regulating smart window device enabled by a mobile app. (c) Automatic response mode of a smart window device designed to operate in response to the surrounding illumination conditions. (d) Demonstration of a smart window that can control the illumination conditions of a model room by switching the scatterer mode on and off. (e) Light intensities at different positions in the room normalized by that at position (1). (f) Pattern encryption feature of a smart window application by coupling the scatterer with Polaroid camera. (g) Next-generation rollable application of a scatterer as an arbitrary projector screen, integrated with the Internet of Things Technology. Reprinted with permission from Refs. [17, 84, 89].

The emerging Internet of Things (IoT) technology can fuel a revolution in scatterer technology, as demonstrated by novel classes of “smart” window systems that can self-regulate their behavior in a user-friendly manner [17, 86, 91]. For example, a stretchable scatterer was devised by combining a film with two digital servomotors, a cadmium sulfide ambient light sensor, and a Bluetooth module (Figure 13a) [17]. The integrated system could read the surrounding illumination conditions from the sensor (Figure 13b) and then automatically respond (transmittance changes) based on the programmed mode: remote manual control and/or a mobile app operation (Figure 13c). When the light was greater than the user-set threshold value of ambient illumination, the smart window device automatically blocked or reflected the incident light by changing to an opaque state and returned to a transparent state when the intensity of the light was lower than the threshold. It should be noted that the response time was less than 1 s, which is difficult to achieve using other types of smart windows.

An equally significant feature of a scattering-based smart window is the capability to moderate the indoor illumination conditions by diffusing the incident light. As shown in Figure 13d, directional incident sunlight freely travels through the transparent scatterer, which is denoted as the “scatterer-off mode,” and can be localized at certain parts of the room. However, when the scatterer is stretched, the transmitted light is diffused by the scatterer that turns to highly opaque, thereby providing relatively uniform illumination across all areas of the room. In particular, positions 2 (wall painting), 3 (picture), and 7 (furniture), where the light cannot effectively reach, become moderate without the assistance of an extra light source (Figure 13e).

Another possible use of a scatterer includes smart optical films designed to meet the esthetic needs of various photographic and/or architectural scenarios. As shown in Figure 13f, a scatterer attached to the lens of a Polaroid camera dramatically affected the appearance of the baby dolls viewed through the films by scattering/blocking most of the visible to NIR light [84]. As a step toward the practical application of a scatterer, a temporary projection screen application was successfully demonstrated to focus a beam-projected image on an exceptionally scalable scatterer (Figure 13g) [17]. Owing to the considerable visible light reflected from the scatterer (a reflectance of ∼40% at 80% strain), the screen-on mode showed the projected image clearly focused on the film. It is noteworthy that this type of wall-type display holds great potential for emerging stretchable or rollable displays that solve the common drawbacks of conventional projection screens, including the spatial constraints and time required to pull out the screen.

This section illustrates some examples of optical applications using a stretchable scatterer in combination with existing window technology and/or as an independent smart optical film. The ability to change the optical modulation on-demand to fulfill a user’s transmittance level preference will be of significant interest for the next generation of urban windows (*i.e.*, climate-adaptive building shells), information encryption, and anti-counterfeiting. Another possible application of the scatterer may include an optical strain sensor which is capable of visualizing the applied strain level and thus quantifying the strain by the optical feature. The concept would be applicable for displaying stress accumulation as a safety sticker with damage indicators for fragile products. Given the current level of the technology, further improvements in the switching reversibility and the chemical/physical resistance of a stretchable film are still needed to accelerate their practical applications. Furthermore, mechanoresponsive scatterers could also be combined with smart windows such as thermo-, photo-, and electro-chromic modes to respond to multiple stimuli in dynamic ways. The controllable resolution of the optical modulation will be much finer through utilizing both the active mode of the mechanoresponsive scatterer and the existing stimuli-triggered optical modulations.

## 6 Conclusion and outlooks

**Figure 14:**
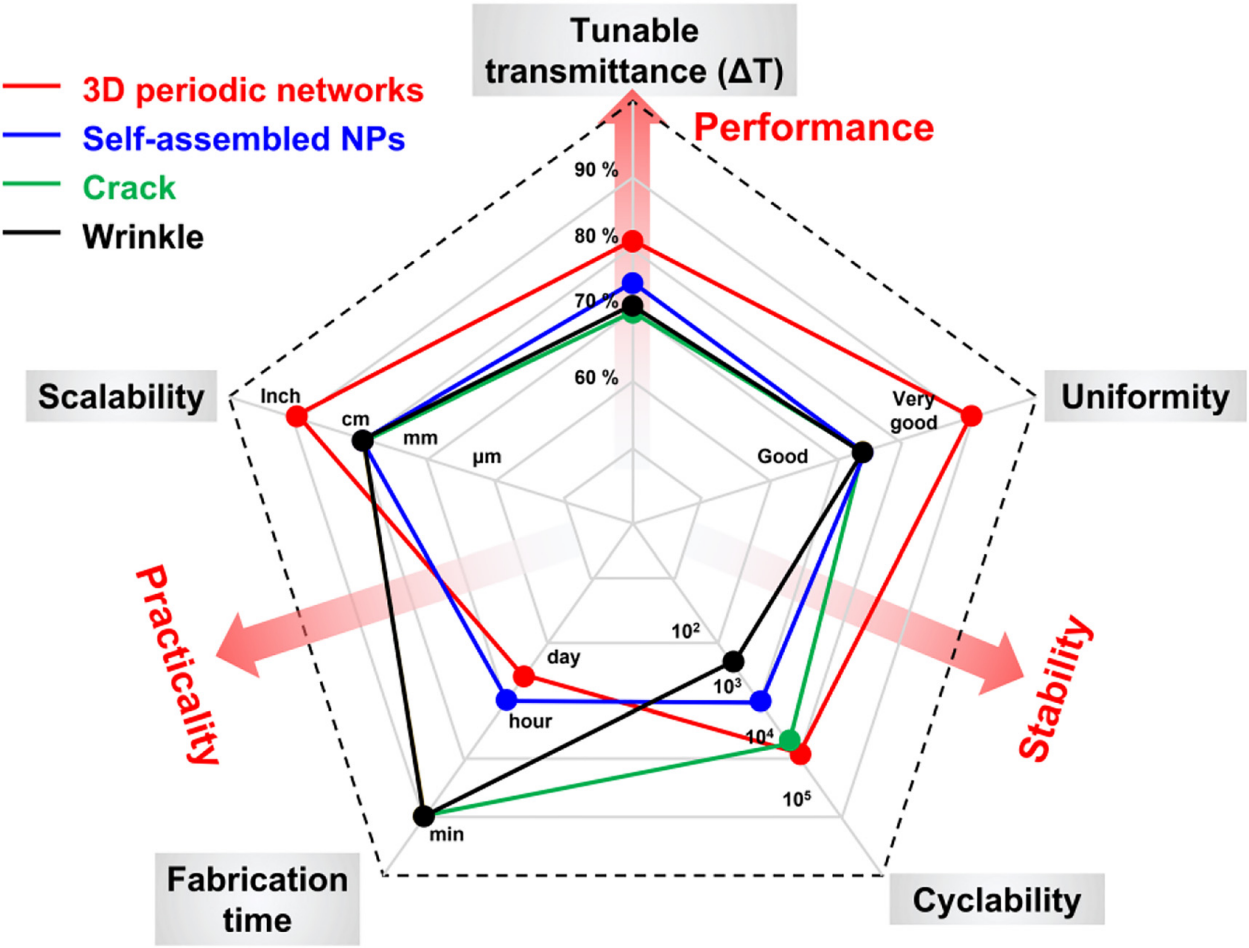
Perspectives for commercialization of high-performance scatterers for practical optical modulating systems.

This review comprehensively examined recent advances in optical scatterers for smart windows driven by mechanical stimuli. This section categorizes the strategies based on the scatterer designs in terms of their performances, thereby dealing with their technical challenges and associated future developments [3, 8, 17, 34, 83], [84], [85, 97]. A brief comparison of representative approaches is presented in Figure 14. The structures of 2D scatterers include cracks and wrinkles, while 3D scatterers are classified as randomly dispersed nanoparticles and 3D periodic networks.

First, 2D scatterers are believed to be more effective for film preparation because they generally require simple manufacturing steps: plasma treatment of the pre-strained silicone and strain release. However, the localized light scattering at the surface generally leads to a relatively insufficient transmittance change (Δ*T* < 60%) due to the intrinsically low optical density, which may limit the full potential of this method for commercialization. Moreover, the unprotected scattering sites of a 2D scatterer at the surface can be vulnerable to external damage, *i.e.*, mechanical deformation and refractive index matching with the surrounding media. Nevertheless, for a straightforward fabrication method, the technologies based on a 2D scatterer are suitable for meeting the engineering demands for optical modulation in patterned regions with controlled shapes and/or more specific areas.

The 3D scatterer has proven its potential in realizing high performance, that is, a transmittance change and cyclability, which are attributed to the high optical density of the embedded scattering sites. However, the proposed fabrication strategies that rely on bottom-up methods such as the self-assembly of synthesized NPs and/or 3D nanopatterning do not eliminate the essential need to control the experimental variables, which require extra care and time. In turn, the fabrication time is more than a few hours, becoming one of the critical bottlenecks for practical use. In this context, it is worthwhile to further diversify the design of a 3D scatterer in a mass-producible manner beyond the lab scale, such as by using roll-to-roll technology, to simultaneously achieve superb uniformity across the entire area. As an example, it is suggested that exceptional scalability could be realized for a 3D scatterer by sequential pixelization and/or the continuous integration of 3D periodic patterns.

Collectively, stringent technical issues remain for mature scatterer technology to open up more possibilities for the commercialization of mechanoresponsive smart windows. Because extensive studies on electrochromic and thermochromic smart windows have established the basis for their commercialization, the technical evolution of scatterers in terms of their practicality, performance, and functionality are strongly needed to transfer their intriguing features to real applications. A better understanding of the underlying physics, along with the development of a new material system, will further broaden the application of scatterers. It is noteworthy that smart window technology has an interdisciplinary research criterion. By exploring the recent advances in scatterers as an emerging topic in the broad scope of materials science, it is envisioned that this review will offer comprehensive interpretations of scatterers to address the growing need for next-generation stretchable and/or rollable displays with many versatile functionalities such as privacy protection, entertainment, and environmental sensing. The great potential of this interdisciplinary inquiry will motivate researchers to develop a new class of scatterers designed to produce a positive global impact.
